# Active prophages as key drivers of microbial adaptation in global soil ecosystems

**DOI:** 10.1128/mbio.00693-26

**Published:** 2026-06-15

**Authors:** Chaofan Ai, Xiang Tang, Haoxiang Han, Yuqi He, Hongbo Zhang, Chen Liu, Hanpeng Liao, Shungui Zhou

**Affiliations:** 1 Fujian Provincial Key Laboratory of Soil Environmental Health and Regulation, College of Resources and Environment, Fujian Agriculture and Forestry University 12449, Fuzhou, China; Shanghai Jiao Tong University, Shanghai, China

**Keywords:** soil, active prophage, auxiliary metabolic genes (AMGs), microbial adaptation

## Abstract

Soils harbor the most complex microbial diversity on Earth, in which bacteria are ubiquitously infected by temperate phages. While integrated prophages often enhance host fitness, active (inducible) prophages are traditionally perceived as “molecular time bombs” due to their intrinsic lysis threat. This dual nature has raised fundamental questions about the true contribution of temperate phages to microbial adaptation and ecosystem stability. To address this gap, we conducted a global-scale integrative analysis by synthesizing 123,207 high-quality bacterial genomes, 183 soil-specific viromic data sets, and 3,749 metagenomes. We established the Global Soil Active Prophage Database (GSAPD), comprising 21,397 high-confidence active prophages, which we found to represent 34.3% of the total soil viral population within our analytical framework. Our comparative genomic analysis reveals that active prophages possess significantly larger genomes and greater genetic complexity compared with their dormant counterparts. Crucially, by mapping phage-encoded auxiliary metabolic genes (AMGs) across diverse biomes, we found that active prophages are disproportionately enriched in key pathways for carbon, nitrogen, and sulfur cycling, as well as specialized resistance mechanisms against heavy metal toxicity. These findings suggest that active prophages act as dynamic reservoirs of functional diversity. We demonstrate that their lytic potential is not merely a survival risk, but a sophisticated mechanism underpinning host environmental adaptation and niche expansion. Ultimately, this study provides a comprehensive global catalog of soil viral pathways and redefines the role of temperate phages as pivotal drivers of microbial evolution and biogeochemical cycling in terrestrial ecosystems.

**IMPORTANCE:**

Soils contain immense microbial diversity, yet the ecological role of temperate phages—especially their active (inducible) forms—remains poorly understood. This study provides the first global-scale assessment of active prophages in soils, revealing that they are widespread and functionally distinct from dormant forms. By building a comprehensive database and integrating multi-omics data, we show that active prophages are enriched in genes linked to key biogeochemical processes and stress resistance. These findings challenge the traditional view of active prophages as purely harmful agents and instead highlight their role as dynamic contributors to microbial function and adaptation. Our work offers new insights into how viruses shape ecosystem processes and provides a valuable resource for future studies on soil microbial ecology and nutrient cycling.

## INTRODUCTION

Soil is the most biodiverse habitat on Earth, harboring approximately 59% of all species and 43% of prokaryotic diversity (1). This extraordinary prokaryotic diversity supports a massive reservoir of soil prokaryotic viruses (i.e., phages), with abundances reaching up to 10^10^ per gram of soil (2, 3), far exceeding those in aquatic ecosystems (4). Unlike aquatic environments, the complex soil matrix—characterized by highly fragmented pore structures and fluctuating moisture levels—imposes unique spatial constraints on phage-host encounters, thereby shaping soil viral ecology and life-history strategies. Based on their life-cycle characteristics, phages are broadly categorized into virulent and temperate types (5, 6). While the former strictly execute the lytic cycle, the latter can alternate their life-cycle between lytic and lysogenic cycles. During the lysogenic cycle, temperate phages integrate into the host genome as prophages to achieve co-replication (7). However, prophages can be triggered by environmental clues to initiate the lytic cycle (8). Numerous studies have demonstrated that prophages confer survival advantages to their bacterial hosts through various mechanisms (e.g., superinfection immunity [9] and metabolic enhancement [10, 11]). Conversely, upon induction, prophages commence the lytic cycle, killing the host cell to release new virions. Indeed, prophage induction has been identified as a primary driver of community collapse in certain scenarios (12). This inherent dual nature raises fundamental questions regarding the actual contribution of prophages to microbial adaptation.

Recent research indicates that prophages can degrade into defective forms (often termed dormant prophages) (13). In this study, we distinguish between two functional states of prophages based on genetic integrity: “active prophages” are defined as those possessing a complete repertoire of genes required for excision and lysis (i.e., lytic-capable), whereas “dormant prophages” (also known as cryptic or defective prophages) refer to those that have lost their inducibility through mutational decay while retaining beneficial traits for the host. Unlike their active counterparts, dormant prophages that derive from genetic mutations lose their inducibility while retaining traits beneficial to host adaptation (14, 15). Intuitively, dormant prophages should possess a symbiotic advantage. However, the fraction of chemically inducible cells in soil varies from 30% to 84% (16). Although such a proxy for lysogen prevalence is constrained by induction efficiency and biases in average burst size, it strongly implies the widespread prevalence and ecological significance of active prophages in soil environments.

Historically, the inducibility of active prophages has been regarded as a threat to host cell survival (17, 18). However, recent studies have challenged this conventional wisdom, positing that the life-cycle transition (lysogenic to lytic) may constitute an adaptive evolutionary strategy. This strategy facilitates community-level advantage at the expense of localized sacrifice under environmental stress. For instance, active prophages have been shown to facilitate the acquisition of antibiotic resistance genes (ARGs) from neighboring bacteria (19). Furthermore, the induction of active prophages serves as a critical pathway for the dissemination of beneficial genes, thereby enhancing host adaptability across the community. Specifically, temperate phages enhance *Pseudomonas aeruginosa* fitness in chronic lung infection (20). Moreover, research has demonstrated that in arsenic-contaminated soils, active prophages enter the lytic cycle and subsequently spread *arsM* gene via secondary infection. These prophages then reintegrate into the bacterial genome to enter a new lysogenic cycle, thereby alleviating arsenite toxicity within the population (21). Despite the potential of both inducible active prophages and permanently integrated dormant prophages to confer adaptive advantages, a fundamental scientific question has long been overlooked: which component plays a more dominant role in driving microbial adaptation. To date, substantial knowledge gaps remain concerning the spatial distribution, genomic structural characteristics, and ecological functional differentiation of active prophages in soils. These limitations hinder a comprehensive mechanistic understanding of phage-host interactions and their evolutionary consequences in complex soil environments.

This study aims to quantify the prevalence of active prophages in wide-ranging soil ecosystems and to elucidate their genetic potential in enhancing microbial adaptation. By integrating 123,207 high-quality bacterial genomes and 3,749 soil metagenomes with 183 soil viromic samples, we utilized DNase treatment and bioinformatic filtering to distinguish active prophages from dormant populations. We further explored the global prevalence of active prophages and compared the genomic architectures and encoded functional genes (associated with nutrient cycling and heavy metal resistance) between active and dormant populations, reflecting the unique distribution pattern in global soils. The present work provides critical evidence for the ecological functioning of active prophages and offers a deeper mechanistic understanding of their roles in driving microbial ecology and biogeochemical processes across diverse soil habitats.

## MATERIALS AND METHODS

### Bacterial genome data collection

To achieve a comprehensive representation of both culturable and unculturable bacterial communities, we integrated high-quality bacterial genomes from pure cultures and metagenome-assembled genomes (MAGs). A total of 94,951 culturable bacterial genomes larger than 1 Mbp (assembled into one chromosome without plasmid) were retrieved from the NCBI GenBank database (January 2024). The quality of these genomes were assessed using CheckM (v1.0.13) (22). Only high-quality genomes with ≥90% completeness and <5% contamination were retained for subsequent analysis. To cover more unculturable bacteria, high-quality bacterial MAGs (completeness >90%, contamination <5%) were collected from the following databases: the Global Microbial Gene Catalog (GMGC) (23), which contributed 46,650 genomes; Genomes from Earth’s Microbiomes (GEM) (24), including 22,565 genomes; and the SMAG catalog (25), contributing 8,525 genomes. In total, 123,207 high-quality bacterial genomes were obtained, comprising 36.9% complete bacterial genomes and 63.1% MAGs, and 36.1% genome sizes (44,521) were predominantly in the range of 2–3 Mb (Table S1). All genomes were classified using the GTDB-Tk classifier with the “classify_wf” workflow (26). *Bacillota* (36.3%), *Pseudomonadota* (29.4%), *Bacteroidota* (14.2%), and *Actinomycetota* (9.0%) are the most dominant bacterial phyla, with a total proportion of 88.9% (Fig. S1).

### Global soil metagenomic data collection

We compiled a global data set of 3,749 soil metagenomes (Table S2) to evaluate prophage and the AMG distribution. Metagenomes were retrieved from NCBI using keywords (“soil,” “land,” “sand,” “metagenomic”) and systematically screened based on metadata descriptions to exclude low-depth samples (<500 Mbp), non-soil environments, or those with explicit host-related sources (e.g., plant endosphere or soil-dwelling animal guts). Sequence quality was assessed via FastQC (v0.12.1) (27), and low-quality reads were trimmed with fastp (v0.24.0) (28) based on default parameters.

### Detection of global soil putative prophage and taxonomic classification

Putative prophage sequences were identified using a combination of two complementary tools, as described previously (29). First, geNomad (v1.7.4), a deep neural network-based viral predictor, detected viral sequences >5,000 bp in bacterial genomes (default parameters) (30). Second, checkV (v1.0.1, database v1.5) (31) was used to remove non-viral sequences, trim host regions, and assess viral sequence quality, retaining sequences with conserved viral signature genes or homology to known phage elements. Genomes with at least one potential prophage were classified as lysogens. Subsequently, the identified prophage sequences were removed duplicates and clustered into viral operational taxonomic units (vOTUs) at 95% average sequence identity with 90% coverage using mmseq2 with the parameters --cov --mode0, identity >95%, coverage >90% (32). These processes yielded 74,102 non-redundant representative prophage vOTUs from the 123,207 bacterial genomes (Table S3). A bacterium was considered a potential lysogen if at least one prophage was successfully detected using the geNomad and CheckV integrated approach. To identify soil prophages, we employed a reference-based mapping strategy rather than direct *de novo* searching within metagenomes. This approach is specifically designed to overcome the challenges posed by high microbial complexity and low sequencing coverage in soil, which often result in fragmented assemblies and the loss of prophage genomic information. Specifically, clean reads from 3,749 soil metagenomes were mapped to the representative prophage sequences with stringent criteria (--percentage_id 0.95, --percentage_aln 0.75) to generate BAM files. Coverage profiles were computed using filtered BAMs (--trim-min 0.10, --trim-max 0.90, --min-read-percent-identity 0.95, --min-read-aligned-percent 0.75, -m mean). This approach identified 63,058 soil prophages from 74,102 non-redundant representative prophage (Table S3). Phage taxonomy was assigned with PhaGCN2 (33), a graph convolutional network tool, using a minimum length of 5,000 bp and referencing the ICTV database (release v4).

### Global soil virome data collection

To evaluate the activity of prophages across diverse soil environments, we analyzed soil extracellular viromes using a combination of in-house samples (*n* = 84) and publicly available data sets (*n* =9 9). In 2023–2024, we collected soil samples from 25 provinces across China, representing various land-use types including fruit farms, vegetable fields, roadsides, and forests. Five random locations were selected for each land-use type, and five replicate surface soil (0–20 cm) samples were collected from each location, yielding a total of 84 composite soil samples (Table S4). All collected soil samples were transported under cryogenic conditions to the laboratory for subsequent analysis, and the fresh soil samples were homogenized through a 2-mm sterile sieve to remove exclude lithic fragments and rhizospheric residues. The homogenate was partitioned into dual subsamples, one of the aliquots was stored at −80°C for DNA extraction, and the other was reserved for soil biochemical properties. Soil viral DNA (*n* = 84) was extracted following previously described methods. Briefly, 10% beef extract and 20 sterile steel beads were added to the soil samples, followed by vortexing and mixing. The mixture was incubated on a shaker at 400 rpm for 30 min, and then centrifuged at 4,700 × *g* for 15 min to obtain the supernatant. The elution process was repeated 2–3 times to ensure complete viral particle recovery. The soil supernatant was then filtered twice using sterile 0.45-μm and 0.22-μm filters to remove bacteria and larger particles, obtaining a soil viral suspension. To ensure the complete removal of contaminating host genomic DNA, the viral concentrates were treated with DNase I (37°C for 50 min). The efficiency of this treatment was validated via PCR amplification of the bacterial 16S rRNA gene using the treated samples as templates. If a visible band was detected by agarose gel electrophoresis, the DNase I concentration or incubation duration was increased until no 16S rRNA amplicons could be generated, thereby confirming the elimination of detectable bacterial DNA. Soil viral DNA was extracted using the TIANamp Viral DNA/RNA Kit (Tiangen, Beijing, China). DNA libraries were prepared using the DNA Library Prep Kit V2 and sequenced on an Illumina Novaseq 6000 platform (Illumina, CA, USA) to generate 150-bp paired-end reads. Additionally, we retrieved 99 soil viromes (Table S4) from the NCBI Sequence Read Archive (SRA) using two inclusion criteria: (i) rigorous depletion of bacterial cells through 0.22-μm membrane filtration of soil lysates, and (ii) enzymatic removal of extracellular DNA fragments via DNase treatment during nucleic acid extraction to eliminate contaminating bacterial DNA fragments. Raw paired-end reads (*n* = 183) were quality controlled using fastp (28) with default parameters.

### Identification of soil active prophages

In the present study, active prophages were defined as those capable of excision from host genomes and release into the soil environment. To determine activity, 63,058 soil prophages were indexed (bowtie2-build) (34), followed by alignment of 123,207 bacterial genomes against the prophage database (bowtie2). BAM files were processed with samtools (35) to isolate pure bacterial genomes (without prophage sequence). The same steps were used to get “pure viral paired-end reads.” Specifically, the pure viral paired-end reads were extracted by aligning reads after quality control against the pure bacterial genomes database (bowtie2). Post-alignment (BAM files), samtools were used to exclude all bacterial-mapped reads, retaining only viral sequences. The pure viral reads were first mapped to prophage using “make” command to create BAM files (--percentage_id 0.95 --percentage_aln 0.75). Filtered BAM files were then used to generate coverage profiles across samples (--trim-min 0.10 --trim-max 0.90 --min-read-percent-identity 0.95 --min-read-aligned-percent 0.75 -m mean). This approach identified 21,397 soil active prophages from 63,058 prophages. To validate the accuracy of the workflow for detecting active prophages, we analyzed 15 lysogenic strains with confirmed mitomycin C-inducibility from our previous study (29). The results showed that the identification accuracy was 100% for active prophages and 92.9% for dormant prophages (Table S5).

### Identification of functional genes in prophages and bacterial hosts

Functional gene prediction was performed on 63,058 prophages and 36,497 associated prophage-free host bacteria (lacking prophage sequences). Briefly, open reading frames (ORFs) on prophages and bacteria were predicted using prodigal and prodigal-gv (36) (a fork of Prodigal meant to improve gene calling for giant viruses and viruses that use alternative genetic codes). The ORFs were annotated for carbohydrate-active enzymes (CAZymes) using dbCAN2 (37). The nutrient cycling associated with nitrogen, phosphorus, and sulfur cycling was identified via DIAMOND (38) alignment of predicted ORFs against specialized metabolic databases: NCycDB (nitrogen cycle database) (39), PCycDB(40) (phosphorus cycle database), and SCycDB (41) (sulfur cycle database) (E-value ≤ 1e−5). Heavy metal resistance genes (MRGs) were predicted by aligning ORFs to the BactMet2D database (42) using blastp (38) (E-value ≤ 10^−5^). Robust identification of potential viral AMGs from metagenomic data remains a major challenge in the field (43). A credible potential AMG must meet two key criteria (44): (i) it should be located between viral genes, with both its upstream and downstream regions flanked by viral hallmark or viral-like genes; and (ii) it should be involved in a cellular metabolic pathway. Therefore, the viral genes were considered high-confidence viral AMGs when viral-like genes were present in their upstream or downstream regions (45). To this end, we used VirSorter2 to detect viral-like genes within 20 genes upstream or downstream of each of the AMGs (46). For comparison, we applied the same approach to quantify AMGs in 654,143 lytic phages from the IMG/VR database (IMG_VR_2022-09-20_7.1) (47) and compared the number of AMGs between prophages and lytic phages.

### Abundance of AMGs and prophages in soils

To investigate the distribution of prophages and AMGs, a non-redundant set of 63,058 prophage sequences was benchmarked against 3,749 global soil metagenomes. Mapping was executed using the CoverM pipeline (v0.61) in contig mode, adhering to previously established protocols (48). Specifically, the relative abundance of each gene was quantified as transcripts per kilobase per million (TPM). Clean metagenomic reads were aligned to the prophage sequences under rigorous criteria (95% identity and 75% alignment coverage) to produce BAM files. Following this, coverage was derived from filtered BAMs (excluding the top and bottom 10% of positions). These TPM-normalized abundance profiles subsequently served as the foundation for both alpha- and beta-diversity assessments.

### Statistical analysis

The R platform were used to statistical analyses (49). To evaluate disparities between active and dormant prophages, data were subjected to either student’s *t*-test or non-parametric alternatives (Wilcoxon rank-sum tests), depending on the normality of the distribution. Relationships between prophage characteristics and bacterial host genomic features were quantified using Pearson correlation. Global distributions of sampling sites were visualized through the “maps” and “ggplot2” packages, with additional data trends illustrated using a diverse suite of “ggplot2”-based visualizations, including violin plots, heatmaps, and stacked bar charts.

## RESULTS AND DISCUSSION

### The establishment of a Global Soil Active Prophage Database

**Fig 1:**
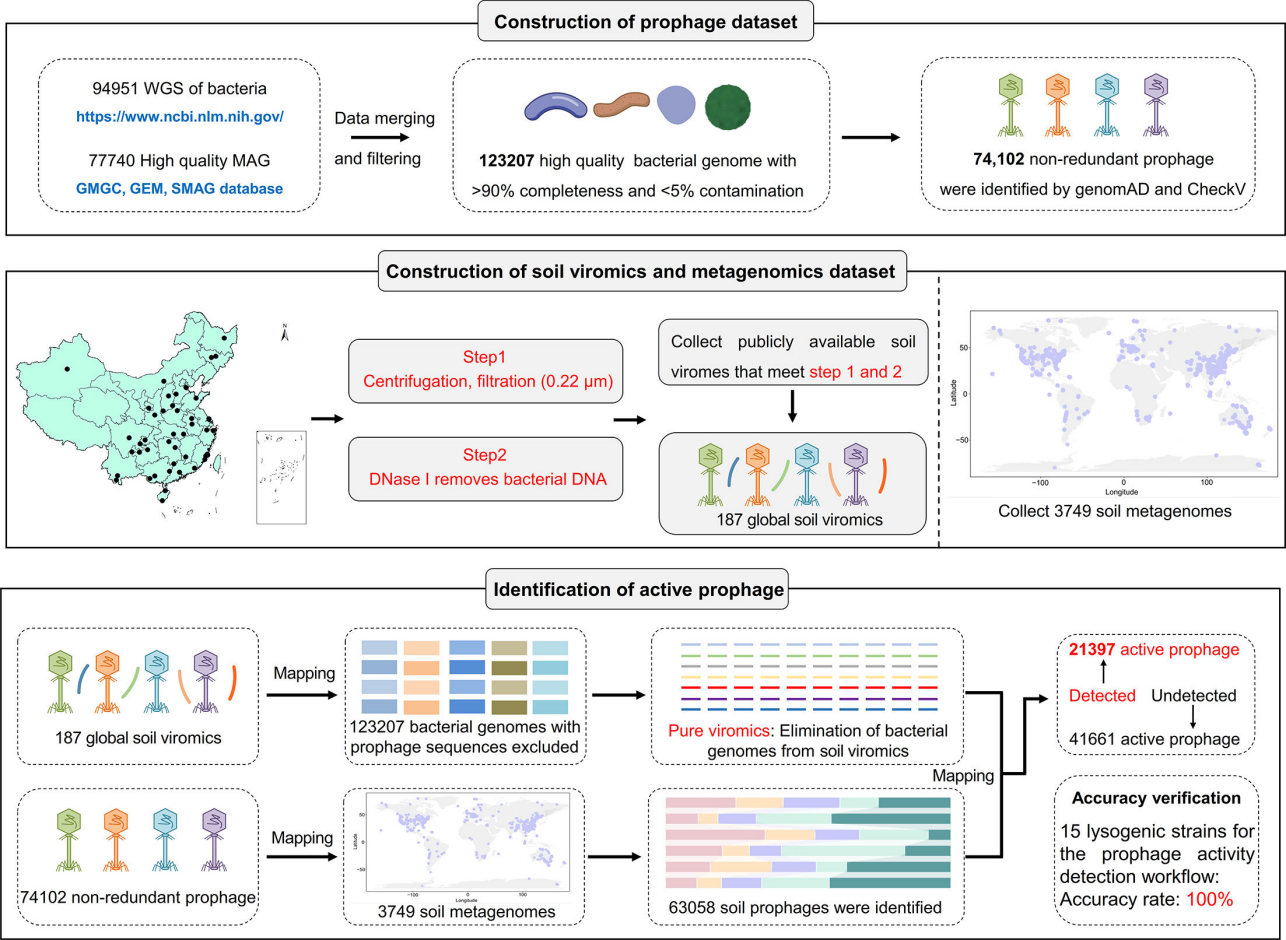
Flow diagram of the methods implemented in the current study. Step 1: Construction of data set. We collected 123,207 high-quality bacterial genomes, predicted 74,102 non-redundant prophage representative sequences. Step 2: Construction of soil viromics and metagenomics data set. We collected 84 soil samples from 24 provinces in China. Soil extracts were centrifuged and passed through a 0.22-μm filter membrane, and DNA enzymes were used to remove residual bacterial DNA during the DNA extraction process before metagenome sequencing, and we collected 103 available viromics sequences that conformed to treatments 1 and 2. In addition, we collected 3,749 global soil metagenomes to assess the community composition and gene abundance of phages in soil environments. Step 3: Identification of active prophage. We mapped the “pure” bacterial genomes into viromics sequences, and eliminated the bacterial genomes to obtain “pure” viral reads. The “pure” virus reads were mapped to 63,058 non-redundant prophage sequences, and the relative abundance of TPM was compared with the viromics data set, if the abundance was greater than 0, it was considered to be an active prophage, and if the abundance was equal to 0, it was considered to be a dormant prophage.

We constructed a global prophage genomic database and systematically assessed its activity within soil environments (Fig. 1). Our analysis initially incorporated 172,691 bacterial genomes from four authoritative databases: NCBI GenBank, GMGC, GEM and the SMAG database. After rigorous quality control (completeness >90% and contamination <5%), 123,207 high-quality bacterial genomes remained, spanning 132 phyla and 4,744 genera. *Pseudomonadota*, *Bacillota*, *Bacteroidota*, and *Actinomycetota* emerged as the predominant taxonomic groups (Fig. S1; Table S1). Utilizing geNomad and CheckV, we identified 79,830 lysogens (64.8% of all genomes) and 223,803 putative prophage genomes within these 123,207 bacterial genomes, consistent with previously reported lysogeny rates of 50%–75% (50, 51). After dereplication (95% ANI and 95% coverage), 74,102 non-redundant putative prophage representative sequences were retained for downstream analysis (Table S2). Comparing our non-redundant sequences against 323,608 prophages in Prophage-DB50 (based on 95% ANI, 95% coverage) revealed that 57.3% of our prophages were novel, thereby significantly expanding the known prophage data set.

To evaluate prophage activity in soil, we designed a multi-step analytical pipeline: (i) we constructed a in-house Chinese soil viromic data set comprising 84 samples from 25 provinces, supplemented with 99 public soil viromic data sets from NCBI (covering China [52], the USA [53, 54], and the UK [55]), all of which underwent DNase treatment to remove host bacterial DNA. (ii) We collected 3,749 public soil metagenomic data sets from NCBI and mapped the 74,102 non-redundant prophages against them, resulting in 63,058 soil-associated prophage sequences; (iii) we established a “clean” bacterial genomic database by removing prophage sequences from the 123,207 high-quality genomes. (iv) Soil viromic reads were aligned against this “clean” bacterial genomic database to filter out residual bacterial DNA. Subsequently, we mapped the 63,058 soil prophages using the purified viromic reads. (v) To validate the accuracy of our pipeline, we applied the four-step analytical workflow to 15 lysogenic strains with known induction activities. The results demonstrated an identification accuracy of 100% for active prophages and 92.9% for dormant prophages. Based on our assessment approach, 21,397 prophages (34.3%) were detected across global soils and classified as active prophages, forming the world’s first global soil active prophage data set. This proportion (34.3%) is significantly higher than the activity levels previously reported in human and murine gut environments (averaging 1.1%–8.9%) (13). This discrepancy primarily stems from two factors: first, methodological differences, as our study leverages high-resolution genomic data and a specialized identification pipeline that enhances detection sensitivity compared with previous high-throughput tools; and second, environmental distinctions, where the high heterogeneity and frequent abiotic stresses characteristic of soil ecosystems may more readily trigger prophage induction compared with more stable, host-associated environments. These were classified as active prophages, forming the world’s first global soil active prophage data set. The remaining 41,661 sequences (65.7%) remained undetected and were consequently categorized as dormant prophages. In terms of prophage genomic quality, CheckV analysis identified 682 complete viral genomes (average length 56.4 kb) and 20,834 high-quality prophage genomes (average length 48.4 kb) within the data set (Fig. S2A). Notably, 45.7% of complete prophages and 49.5% of high-quality prophages were identified as active, both significantly exceeding the overall activity rate (34.3%); conversely, only 20.7% of low-quality prophages exhibited activity (Fig. S2B). These findings suggest that our analytical strategy may still underestimate the true prevalence of active prophages in soil ecosystems. This potential underestimation likely stems from two primary factors. First, the technical limitations of metagenomic assembly: low-quality prophages, often characterized by fragmented or incomplete sequences, may lack the diagnostic genomic features (e.g., specific attachment sites or essential structural genes) required for confident activity identification. Second, the inherent bias of induction methods; for instance, while mitomycin C is a standard chemical inducer, it may fail to trigger the excision of prophages that respond to alternative environmental cues, such as nutrient fluctuations or SOS-independent pathways.

### Active prophages are unique groups

**Fig 2:**
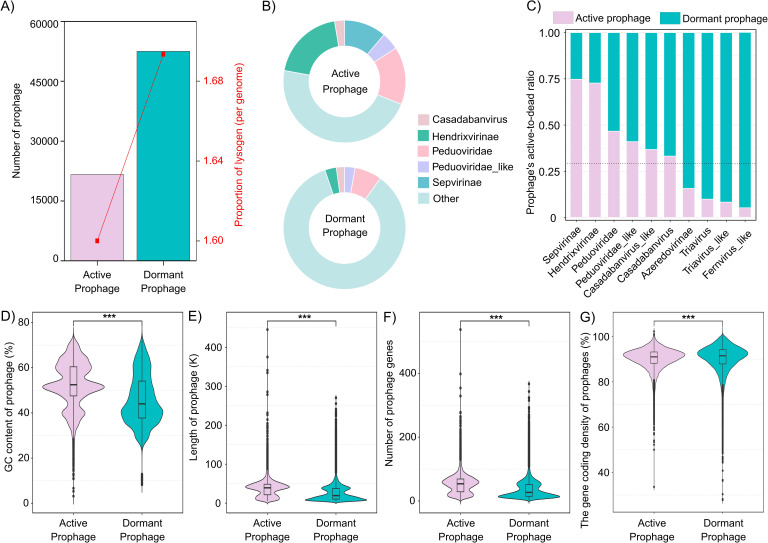
Comparison of genomic features of active and dormant prophage. (**A**) The number (bar plot) and proportion (line plot) of active and dormant prophage. (**B**) The taxonomic assignment of active and dormant prophage. (**C**) The proportion of active and dormant prophage at family level. Violin plot represents the (**D**) genome size, (**E**) GC content, and (**F**) number of prophage genes of active and dormant prophage. Student’s *t*-test was applied when data followed a normal distribution, whereas non-parametric alternatives (Kruskal–Wallis and Wilcoxon tests) were used otherwise, and the significance levels are indicated by *** (*P* < 0.001). Box plots show the 25th–75th percentiles, whiskers indicate the minimum and maximum values, and the midline represents the median.

Active and dormant prophages are distributed across distinct bacterial taxonomic groups. Specifically, 21,397 active prophages were identified across 13,407 lysogens, with an average of 1.6 active prophages per lysogen, spanning 46 phyla and 1,142 genera. Among these, *Pseudomonadota* (79.6%), *Bacillota* (7.9%), and *Actinomycetota* (6.2%) emerged as the primary host taxa, accounting for 93.7% of all active hosts (Fig. 2A; Fig. S3A). In contrast, 41,661 dormant prophages were distributed across 76 phyla and 2,398 genera, averaging 1.5 per lysogen, with *Bacillota* (46.3%), *Pseudomonadota* (26.3%), and *Bacteroidota* (12.4%) as the dominant hosts (Fig. 2A; Fig. S3B). Furthermore, the prevalence of active prophages varied substantially across bacterial phyla. Among the top five host phyla, only *Pseudomonadota* (60.8%) and *Actinomycetota* (36.3%) exhibited active prophage ratios exceeding the overall average (34.3%, Fig. S4A). Notably, at the genus level, active prophages were significantly enriched in genera containing known opportunistic pathogens, such as *Pseudomonas*, *Escherichia*, and *Klebsiella*, representing 15.4%, 12.5%, and 9.0% of all active prophage hosts, respectively. The active prophage carriage rates within these genera far surpassed the global average; for instance, in *Pseudomonas* and *Enterobacter*, the rates reached as high as 89.9% and 88.2%, respectively (Fig. S4B). These results indicate that active prophages exhibit pronounced phylogenetic host preferences, particularly within potentially pathogenic groups, reflecting their potentially unique roles in host ecological adaptation.

We subsequently compared the phylogenetic distribution and genomic characteristics of active and dormant soil prophages. Using PhaGCN2, 73.3% (46,525 of 63,508) of prophages were taxonomically assigned. The 21,397 active prophages were classified into 151 viral families, predominantly from *Hendrixvirinae* (19.7%), *Peduoviridae* (15.3%), and *Sepvirinae* (11.3%) (Fig. 2B). Conversely, the primary dormant prophages belonged to *Peduoviridae* (7.7%), *Azeredovirinae* (5.1%), and *Triavirus* (3.9%) (Fig. 2B). Moreover, activity ratios differed significantly across viral families; for example, *Sepvirinae*, *Hendrixvirinae*, and *Peduoviridae* showed activity rates of 74.8%, 72.3%, and 46.7%, respectively—all markedly higher than the average of 34.3% (Fig. 2C). This suggests distinct phylogenetic distribution patterns between active and dormant prophages. Regarding genomic features, active prophages possessed significantly higher GC content (1.1-fold, 52.9% vs 46.5%, *P <* 0.05, Fig. 2D) and longer genomes (1.4-fold, 38.3 kb vs 27.8 kb, *P <* 0.05, Fig. 2E) compared with dormant ones. The significantly longer genomes observed in active prophages compared with dormant ones likely reflect distinct evolutionary trajectories. Active prophages may maintain larger genomes to accommodate a diverse array of AMGs and regulatory elements that provide immediate fitness advantages to their hosts, thereby ensuring the preservation of the viral sequence through positive selection. In contrast, the smaller size of dormant prophages may be a hallmark of gene degeneration and reductive evolution. As dormant prophages lose their capacity for induction and vertical transmission becomes their sole strategy, they likely experience weaker purifying selection on viral replication genes, leading to progressive gene loss and genome attrition over evolutionary timescales. Although active prophages harbored more protein-coding genes (1.4-fold, 53.0 vs 38.1, *P <* 0.05, Fig. 2F), their gene-coding density was slightly but significantly lower than that of dormant prophages (90.23% vs 90.42%, *P <* 0.05, Fig. 2G). This suggests that active prophages may adopt a relatively more expansive genomic architecture, potentially to accommodate complex regulatory elements required for their active lifecycle, whereas dormant sequences exhibit a higher degree of genomic streamlining. A trend consistent across the five most abundant viral families (Fig. S5). These similarities likely result from long-term phage-host interactions leading to convergent genomic properties. Indeed, hosts of active prophages also displayed larger genomes (4.6 Mb vs 3.4 Mb, Fig. S6A), higher gene counts (4,425.6 vs 3,171.1, Fig. S6B), and elevated GC content (55.2% vs 47.2%, Fig. S6C) relative to dormant prophage hosts. Furthermore, significant positive correlations were observed between prophages and their host bacteria regarding GC content (R = 0.95, *P <* 0.05, Fig. S7A), genome length (R = 0.22, *P <* 0.05, Fig. S7B), and gene count (R = 0.19, *P <* 0.05, Fig. S7C). Together, these findings demonstrate that active and dormant prophages represent two distinct groups, with the active component possessing larger genomes and a greater repertoire of genes.

### Active prophage genomes encode more AMGs relative to the dormant fraction

**Fig 3:**
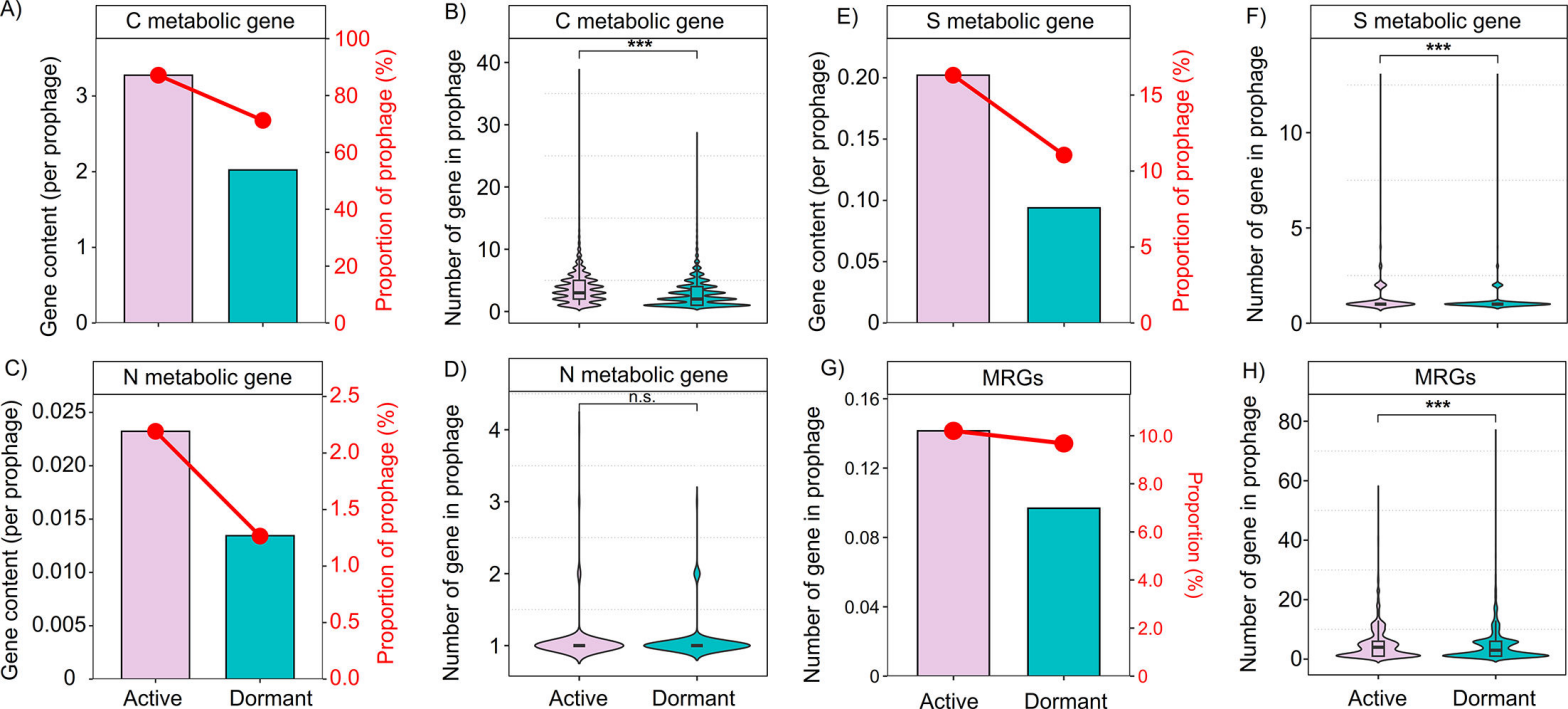
Distribution of genes related to the carbon (carbohydrate-active enzymes), nitrogen, and sulfur metabolism genes and metal resistance genes (MRGs) in active and dormant prophage genomes. Panels **A** and **B** represent the number and proportion of carbon metabolism genes encoded by prophage genomes. Panels **C** and **D** represent the number and proportion of nitrogen metabolism genes encoded by prophage genomes. Panels **E** and **F** represent the number and proportion of sulfur metabolism genes encoded by prophage genomes. Panels **G** and **H** represent the number and proportion of MRGs encoded by prophage genomes. Box plots show the 25th–75th percentiles, whiskers indicate the minimum and maximum values, and the midline represents the median. The significance levels are indicated by n.s. (*P* > 0.05) and *** (*P* < 0.001). Student’s *t*-test was applied when data followed a normal distribution, whereas non-parametric alternatives (Kruskal–Wallis and Wilcoxon tests) were used otherwise.

To compare the functional roles of active and dormant prophages in biogeochemical cycling, we systematically characterized their AMGs related to carbon, nitrogen, and sulfur metabolism, as well as heavy metal resistance. A critical challenge in AMGs identification is distinguishing true viral genes from misclassified host functional genes. To minimize this risk, the presence of viral structural or hallmark genes flanking an AMG is widely accepted as a robust criterion for reliable identification. In our data set, 83.1% of identified AMGs were flanked by viral genes on both sides, while 16.7% had a viral gene on one side (Fig. S8). These findings provide strong evidence that the AMGs identified here for carbon, nitrogen, and sulfur metabolism and heavy metal resistance are authentic viral genes rather than host contamination. Across all 63,058 soil prophage genomes, we identified 2,722,056 ORFs (averaging 43.2 ORFs per prophage), with active prophages encoding 1.4 times more ORFs than dormant prophages. Notably, both active and dormant prophages encoded significantly more genes than virulent phages (*P <* 0.05, Fig. S9). Regarding carbon metabolism, carbohydrate-active enzymes (CAZymes) were detected in 76.7% (48,339 of 63,058) of the prophages. Specifically, 87.1% (18,645 of 21,397) of active prophages encoded CAZyme genes, significantly higher than the 74.3% (29,694 of 41,661) observed in dormant prophages (Fig. 3A). Furthermore, active prophages carried a significantly higher number of CAZyme genes per genome (3.8 vs 2.8, *P <* 0.05, Fig. 3B). Both groups encoded all six major CAZyme classes: glycoside hydrolases (GHs), glycosyltransferases (GTs), carbohydrate esterases (CEs), polysaccharide lyases (PLs), auxiliary activities (AAs), and carbohydrate-binding modules (CBMs). Active prophages primarily encoded GHs (77.3%), GTs (11.6%), and CBMs (7.3%), with GH24 (23.6%), GH23 (20.8%), and GT2 (6.1%) being the most prevalent families (Fig. S10). In contrast, dormant prophages mainly carried GHs (70.1%), CBMs (16.4%), and GTs (10.9%), predominantly from the GH23 (21.8%), GH24 (13.1%), and CBM50 (11.5%) families. Overall, prophages extensively harbor CAZyme genes, with active prophages encoding more carbon metabolism genes.

In terms of nitrogen metabolism, 1.6% (996 of 63,058) of the prophages encoded 1,057 nitrogen metabolism genes. While the total number of nitrogen metabolism genes did not differ significantly between the two groups (Fig. 3C), the proportion of active prophages carrying these genes was higher than that of dormant prophages (2.3% vs 1.3%, Fig. 3D). These genes spanned five categories: organic degradation and synthesis, assimilatory nitrate reduction, dissimilatory nitrate reduction, nitrogen fixation, and denitrification. Active prophages mainly focused on organic nitrogen degradation/synthesis (75.1%) and dissimilatory nitrate reduction (13.7%), whereas dormant prophages were primarily involved in organic nitrogen degradation/synthesis (88.2%) and assimilatory nitrate reduction (4.2%) (Fig. S11).

Regarding sulfur metabolism, 8,237 genes were detected in 11.1% (6,969 of 63,058) of the prophages. The frequency of active prophages carrying sulfur metabolism genes was 1.5 times that of dormant ones (16.3% vs 11.1%, Fig. 3E), and their average gene count was 2.3 times higher (0.16 vs 0.07, *P <* 0.05, Fig. 3F). These genes represented eight major sulfur transformation pathways. Active prophages predominantly encoded genes for organic sulfur transformation (39.8%), the link between inorganic and organic sulfur transformation (33.8%), and assimilatory sulfate reduction (14.3%), with *mdh* (12.9%), *cysE* (10.9%), and *cysK* (8.3%) as the dominant genes (Fig. S12). On the contrary, dormant prophages showed a 2.3-fold higher proportion of genes for assimilatory sulfate reduction compared with active prophages.

Finally, we compared heavy metal resistance genes (MRGs) based on the BacMet database. We found that 8.9% (6,216 of 63,058) of the prophages carried 8,444 MRGs. The carriage rates for active and dormant prophages were 10.2% and 9.7%, respectively (Fig. 3G), but active prophages carried significantly more MRGs per genome (0.14 vs 0.10, *P <* 0.05, Fig. 3H). Both groups showed similar resistance profiles across 19 metal types, dominated by multi-metal resistance (43.6% vs 44.0%), followed by copper (Cu), arsenic (As), zinc (Zn), and iron (Fe) (Fig. S13). In conclusion, compared with dormant prophages, active prophages are enriched with carbon, nitrogen, and sulfur metabolism genes and MRGs. These findings suggest that active prophages possess a higher genomic potential to modulate soil biogeochemical cycling and support host adaptation to environmental stress.

### Active prophages dominate the functional potential and distribution of the soil prophage

To further elucidate the metabolic potential of active and dormant prophages in soils, we first investigated the global distribution patterns of prophage communities based on 3,749 soil metagenomes. Overall, 85.1% of prophages (63,058 of 74,102) were detectable across global soil metagenomes, with an average of 525.7 prophages identified per metagenomic sample. Across all soil metagenomes, detection frequencies varied substantially among viral families. In total, 193 viral families were detected, with most families exhibiting low detection frequencies (0%–10%, 75 families). In contrast, 30 viral families showed detection frequencies exceeding 90%, including *Hendrixvirinae*, *Casadabanvirus*, and *Peduoviridae*, each detected in 99.9% of global soil samples (Fig. S14). Viral family abundances also differed markedly among soils. *Microviridae* (20.9%), *Casadabanvirus_like* (10.3%), *Peduoviridae* (8.3%), *Hendrixvirinae* (6.2%), and *Casadabanvirus* (5.8%) were the dominant families, together accounting for 51.5% of the total prophage abundance (Fig. S15). Collectively, these results indicate that prophages are ubiquitously distributed across global soil environments. We next compared the biogeographic distribution patterns of active and dormant prophage communities. Active prophages exhibited a broader distribution across soils: on average, 688.5 active prophages were detected per soil sample, whereas only 363.1 dormant prophages were detected per sample. Moreover, active prophages showed significantly higher global soil abundance (*P* < 0.05, Fig. S16A) and α-diversity than dormant prophages, as indicated by both the Richness index (*P* < 0.05, Fig. S16B) and the Shannon index (*P* < 0.05, Fig. S16C). Active prophage communities were characterized by pronounced dominance patterns, whereas dormant prophage communities were more evenly distributed. Specifically, the dominant viral families of active prophages were *Microviridae* (25.8%), *Casadabanvirus_like* (10.9%), and *Peduoviridae* (7.6%), with the top 10 viral families, accounting for 78.0% of the total abundance (Fig. S17A). In contrast, the dominant families of dormant prophages were *Peduoviridae* (10.9%), *Casadabanvirus_like* (7.6%), and *Dolichocephalovirinae_like* (7.3%), together accounting for only 51.6% of the total abundance (Fig. S17B). These results demonstrate pronounced differences in community diversity and composition between active and dormant prophages in global soils.

**Fig 4:**
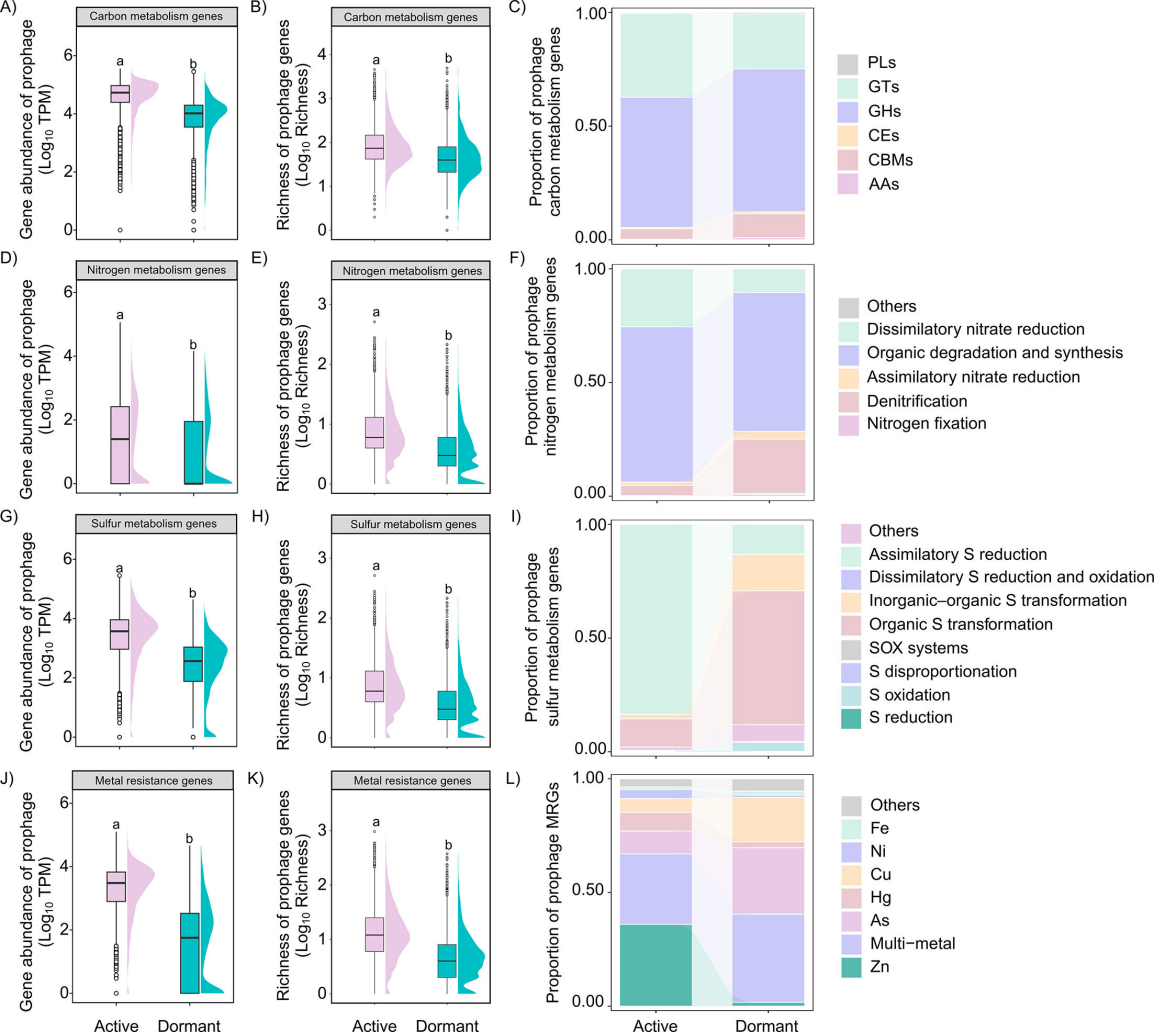
Distribution of active and dormant prophage genes related to carbon, nitrogen, sulfur metabolism, and metal resistance genes (MRGs) in soil (*n* = 3,749). The first-row plots present the carbon metabolism (**A**) gene abundance, (**B**) richness index, and (**C**) composition of active and dormant phages in the soil. The second-row plots present the nitrogen metabolism (**D**) gene abundance, (**E**) richness index, and (**F**) composition of active and dormant phages in the soil. The third-row plots present the sulfur metabolism (**G**) gene abundance, (**H**) richness index, and (**I**) composition of active and dormant phages in the soil. The fourth-row plots present the sulfur metabolism (**J**) gene abundance, (**K**) richness index, and (**L**) composition of active and dormant phages in the soil. Box plots show the 25th–75 th percentiles, whiskers indicate the minimum and maximum values, and the midline represents the median. Different lowercase letters indicate statistically significant differences at *P* < 0.05. Student’s *t*-test were applied when data followed a normal distribution, whereas non-parametric alternatives (Kruskal-Wallis and Wilcoxon tests) were used otherwise.

To explore functional differences between active and dormant prophages, we further examined the soil distribution patterns of their AMGs. Overall, 36.2% of prophage ORFs (991,696 of 2,715,870) were detectable across global soil metagenomes, with an average of 1,951.8 prophage functional genes identified per metagenomic sample. When stratified by prophage state, AMGs encoded by active prophages were more prevalent in soils, with a detection rate of 46.5% across all soil metagenomes, compared with only 24.8% for dormant prophages. In addition, the abundance of AMGs encoded by active prophages was significantly higher than that of dormant prophages (*P < 0.05*, Fig. S18). We then focused on the distribution patterns of prophage-encoded nutrient cycling genes and MRGs in soils. For carbon metabolism, 53.1% (82,074 of 154,332) of prophage-encoded carbon metabolism genes were detectable in soils. Among these, GHs, GTs and CBMs were the dominant prophage-encoded CAZyme (pCAZyme) genes, accounting for 63.4%, 23.1%, and 10.2% of the total, respectively (Fig. S19). Carbon metabolism genes encoded by active prophages exhibited significantly higher soil detection rates (63.1% vs 44.6%), abundances (4.6 vs 3.8, log_10_[TPM], *P* < 0.05; Fig. 4A), and α-diversity (5.8 vs 5.0 based on the Richness index, *P* < 0.05; Fig. 4B) than those encoded by dormant prophages. The dominant carbon metabolism gene categories also differed between the two types of prophages. For instance, GTs accounted for 36.9% of all CAZyme genes encoded by active prophages—1.5 times higher than in dormant prophages (24.1%)—whereas CBMs comprised only 7.3% of active pCAZymes but 16.4% of dormant prophage pCAZymes (Fig. 4C). Regarding nitrogen metabolism, 75.4% (796 of 1,056) of genes were detected. Active prophages exhibited higher detection rates (85.5% vs 66.4%), abundance (Fig. 4D), and diversity (Fig. 4E) for N-metabolism genes compared with dormant prophages. Notably, dissimilatory nitrate reduction (primarily the *nrfC* gene) was the dominant pathway for active prophages (25.5% vs 10.4% in dormant), whereas denitrification was more prominent in dormant prophages (23.8% vs 4.5% in active) (Fig. 4F). For sulfur metabolism, active prophages also showed significantly higher detection rates, abundance, and diversity (Fig. 4G and H). Assimilatory sulfur reduction was the dominant pathway in active prophages (83.5%), while organic sulfur transformation prevailed in dormant ones (58.8%, Fig. 4I). Similarly, MRGs in active prophages showed superior detection rates (78.6% vs 57.0%), abundance (Fig. 4J), and diversity (Fig. 4K). Furthermore, gene compositions shifted significantly; the proportion of Zn-related MRGs in active prophages was 21.2 times higher than in dormant ones, while Cu-related genes were only 31.3% as prevalent (Fig. 4L). In summary, the AMGs of prophage are widely distributed in soil, with active prophages maintaining a significant advantage in functional gene abundance and diversity, suggesting they fulfill distinct ecological roles compared with dormant prophages.

## DISCUSSION

Our study provides a comprehensive resolution to the fundamental question of whether active or dormant prophages play the relative roles in soil microbial adaptation. By establishing the GSAPD, we demonstrate that active prophages are not merely “molecular time bombs” but represent significant reservoirs of functional diversity within the soil virome. Our findings reveal that active prophages represent a substantial fraction (34.3%) of the soil viral community and possess a significantly more complex genomic architecture compared with their dormant counterparts. Crucially, the disproportionate enrichment of AMGs related to carbon, nitrogen, and sulfur cycling, alongside heavy metal resistance genes within the active population, indicates that their lytic potential is closely linked to a high capacity for metabolic augmentation. However, it is important to note that the presence of these genes does not necessarily indicate their expression or functional activity, and thus their ecological effects should be interpreted with caution. These results collectively suggest that the dynamic “inducibility” of active prophages facilitates a potentially more rapid and robust dissemination of adaptive traits than the static presence of dormant prophages. Consequently, we propose that the life-cycle transition of active prophages is an important contributor to microbial functional evolution and community-level resilience across diverse soil biomes.

One of our primary findings is that the AMGs encoded by active prophages are markedly superior to those encoded by dormant prophages in both abundance and functional diversity at the macroecological scale. This necessitates a reassessment of the ecological role of active prophages in host bacteria. From a phage–host interaction perspective, this pattern may arise from the following mechanisms. First, active prophages retain the capacity to enter the induction cycle and release viral particles, rendering them highly mobile genetic elements rather than static sequences embedded within host chromosomes. Given the spatial and temporal heterogeneity of soil environments, active prophages may be under strong selective pressure to acquire AMGs that enhance host fitness to ensure their own persistence under diverse environmental stresses, while low-frequency prophage induction results in localized host cell death, it facilitates widespread gene dissemination at the population level (19, 56, 57). In contrast, dormant prophages, which no longer enter the lytic cycle, experience weaker purifying selection and are therefore more prone to mutation accumulation and functional gene loss. Second, at the population level, the enrichment of AMGs in active prophages may represent a community-level defense strategy. Under low-frequency induction, the induction of prophages in a subset of individuals causes localized host cell death. However, when the released progeny phages carrying AMGs reinfect and lysogenize surrounding conspecific bacteria, they facilitate the rapid dissemination of beneficial genes at the population level. Recurrent cycles of induction and reinfection further reinforce the AMG advantage of active prophages and expand host functional diversity at the population scale. The strong correlations observed between prophage and host GC content, genome length, and gene number support this hypothesis. Our previous work provides empirical support for this mechanism: in flooded arsenic-contaminated soils, partial induction of prophages led to infection of new hosts and rapid dissemination of the *arsM* gene within soil microbial communities (21). Similarly, in paddy soils amended with exogenous Na₂HAsO₄, lysogenic prophages were observed to enrich genes involved in arsenic oxidation and phosphorus co-metabolism (58). The present study further demonstrates, at both genomic and global soil scales, that active prophages are a critical component enabling hosts to acquire enhanced heavy metal resistance gene repertoires. In this study, the MRGs as a representative model for environmental adaptation, as heavy metal stress is a ubiquitous and persistent selective pressure in diverse soil ecosystems, making MRGs a clear proxy for assessing how prophages enhance host fitness.

**Fig 5:**
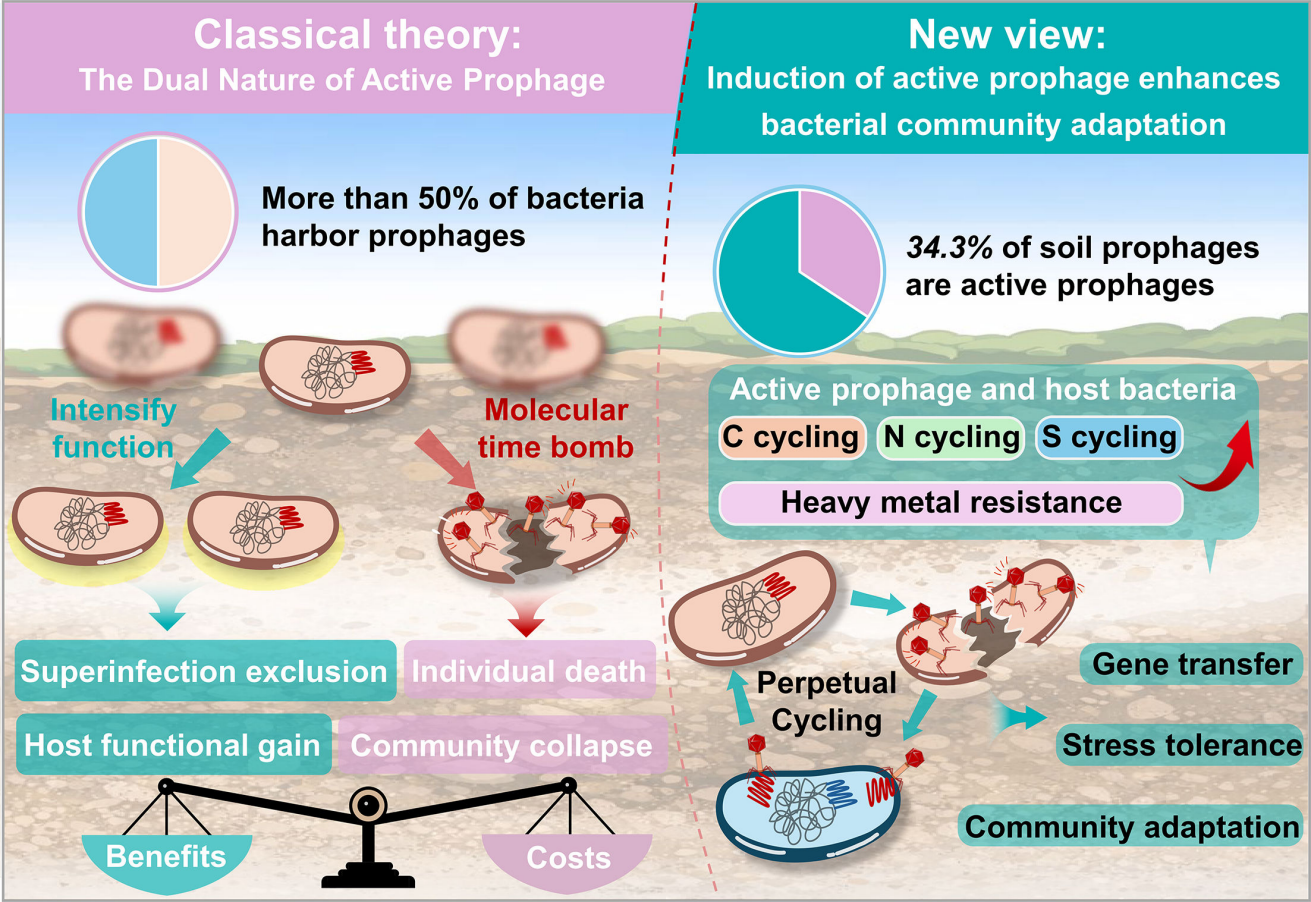
Conceptual model for the role of active prophages in enhancing bacterial community fitness in soil. The left panel illustrates the classical theory of active prophages, conceptualized as a balance scale to emphasize their dual nature. On one side, prophages provide immunity against superinfection and enhance host metabolic functions through auxiliary metabolic genes (AMGs). On the other side, the induction of active prophages is regarded as a “molecular time bomb,” which can trigger host cell lysis, leading to individual mortality and potentially destabilizing the microbial community. The right panel presents the perspective emerging from this study while retaining the environmental context shown in the background. Active prophages constitute 34.3% of the total soil prophage pool and, at the population level, act as “genetic reservoirs” that facilitate the horizontal transfer of functional genes (e.g., those involved in C, N, and S cycling, as well as heavy metal resistance). This mechanism enhances host tolerance to environmental stress and ultimately improves the overall ecological fitness and adaptive capacity of the bacterial community.

In addition to metal resistance, active prophages may also facilitate host acquisition of nutrient cycling genes, potentially enabling occupation of higher ecological niches. Specifically, carbon, nitrogen, and sulfur metabolism genes encoded by active prophages exhibit significantly higher detection frequencies, abundances, and diversities in soils than those encoded by dormant prophages. This pattern may reflect the inducible nature of active prophages, which could enhance their ecological responsiveness and allow both prophages and hosts to gain competitive advantages more rapidly under nutrient-limited conditions. These findings are consistent with previous studies showing that, in deep-sea sediments, *Pseudomonas* prophages can be induced under chitin-rich conditions, promoting host chitin metabolism via the dissemination of chitinase-encoding genes (59). Similarly, in carbon-limited soils, prophages can enhance host interactions and enrich genes involved in organic matter degradation (60). In contrast, dormant prophages appear less capable of providing effective countermeasures under fluctuating environmental conditions (16), which may contribute to distinct preferences in nutrient-cycling AMGs between active and dormant prophages. Specifically, beyond the dominant GHs families, GTs encoded by active prophages represent the predominant carbon metabolism gene category in soils. These enzymes participate in the synthesis of polysaccharides, glycolipids, and glycoproteins, thereby enhancing host cell wall construction, glycosylation modifications, and complex carbon utilization (61)—an ecological strategy that could promote prophage replication and dissemination. In contrast, the significant enrichment of CBMs in dormant prophages suggests a strategy focused on enhancing host substrate binding and resource localization rather than increasing metabolic flux (60, 62). This potential slow-carbon-utilization approach may be advantageous for host survival in nutrient-poor or stable soil microenvironments, supporting the long-term lysogenic maintenance of dormant prophages. Distinct strategies are also evident in nitrogen and sulfur metabolism. Active prophages preferentially encode genes for dissimilatory nitrate reduction, a pathway capable of rapidly supplying metabolically active hosts with assimilable nitrogen (NH₄^+^), thereby potentially enhancing host growth and adaptability. In contrast, dormant prophages are enriched in denitrification genes, consistent with their prevalence in anoxic, low-energy soil environments where denitrification represents a low-cost anaerobic respiratory strategy (63). Similarly, the enrichment of low-cost organic sulfur transformation genes in dormant prophages may facilitate host persistence under oligotrophic conditions (64), whereas active prophages preferentially encode assimilatory sulfate reduction genes. This pathway supports energy-intensive sulfate assimilation and burst synthesis of sulfur-containing amino acids (65), potentially representing a maximal investment in host—and prophage—replication efficiency. These functional distinctions are further reflected in global soil prophage community structure. Active prophage communities exhibit a pronounced “winner-takes-all” pattern, with the top 10 viral families accounting for 78.0% of total abundance, whereas dormant prophage communities are more evenly distributed, with the top 10 families contributing only 51.6%. Notably, this dominance pattern is strongly correlated with the relative abundance of their corresponding host bacteria (*R* = 0.87, *P* = 0.002, Fig. S20), suggesting that the ecological success of active prophages may be closely linked to the population dynamics of their hosts. Naturally inducible active prophages thus appear to form a small number of highly successful ecotypes in soils, potentially achieving greater global dominance through higher lysis–infection efficiency or superior environmental adaptability. Similar dominance patterns have been observed for lytic phages in marine ecosystems (66). Overall, our results suggest that active prophages play a more important role in enhancing host adaptability than dormant prophages, challenging the view of them merely as molecular time bombs poised to lyse their hosts. Compared with the ostensibly “safe” dormant prophages, active prophages may possess greater gene dissemination potential and environmental responsiveness, enabling them to enhance population-level adaptability under fluctuating soil conditions (Fig. 5).

Despite revealing global-scale patterns of active soil prophages, this study has several technical limitations. First, by refining the identification of active prophages, our approach transcends the constraints of host culturability and phage visibility. Furthermore, it bypasses the potential inaccuracies of methods dependent on static genomic signatures, including integrase detection. However, our assessment of active prophages was restricted to those inducible by spontaneous induction or mitomycin C treatment, which cannot fully capture the complex and transient induction mechanisms operating in natural environments. Consequently, the global proportion of active prophages may still be underestimated. We provide a potential solution: by assembling large-scale data sets of experimentally validated inducible prophages, deep learning models could be developed to identify sequence-specific signatures of activity, enabling environment-independent prediction of prophage activity and reducing misclassification caused by environmental heterogeneity. Second, the actual phenotypic contributions of bioinformatically identified AMGs within host cells require experimental validation. To address these limitations, the integration of integrating metatranscriptomics or single-cell approaches would allow direct assessment of the expression and metabolic contributions of active and dormant prophages at the molecular level, providing more robust evidence of prophage activity.

This study provides initial evidence that active prophages confer clear adaptive advantages to their hosts. First, focusing on extreme or stressed environments—including mine tailings, saline soils, and nutrient-poor settings, such as deep-sea sediments—will be essential for future research. Such studies will provide critical insights into quantifying the specific contributions of active prophages to host bacterial adaptation under environmental stress. Second, the application of appropriate tracer techniques will be critical for tracking active prophage-mediated gene flow and host lineage specificity in complex soil matrices. Finally, we call for a collective effort to expand active prophage databases. Integrating more comprehensive genomic signatures will enable a deeper mechanistic understanding of prophage-host interactions and their multidimensional responses to environmental shifts. Beyond basic ecological insights, such efforts will provide the essential theoretical foundation for leveraging prophages in soil remediation and sustainable agriculture. However, from the perspective of soil health and biosafety, the high dissemination potential of active prophages must be viewed as a double-edged sword. For instance, the high proportion of active prophages observed in human pathogenic bacteria suggests a specialized mechanism for rapid genomic diversification. Such high inducibility may facilitate the “active” mobilization of virulence factors and antibiotic resistance genes (ARGs) across the soil–human interface, thereby posing a unique biosafety risk. Consequently, while active prophages facilitate the spread of beneficial nutrient-cycling genes and MRGs, they may also act as vectors for the propagation of harmful elements (such as ARGs), potentially exacerbating the spread of multi-drug resistance within soil environments. Future research must therefore balance the adaptive benefits of prophage-mediated gene flow against the ecological risks posed by the mobilization of ARGs. Integrating these insights with advanced biotechnological strategies (e.g., genome editing) could facilitate the development of targeted approaches to enhance soil ecosystem functions while rigorously mitigating associated biosafety risks (67).

## Data Availability

The data generated in this study are provided in the Supplementary Information. Source data are provided with this paper. The Global Soil Active Prophages Database (GSAPD) generated in this study is available in the Zenodo repository at https://zenodo.org/records/19510112.

## SUPPLEMENTAL MATERIAL

Supplemental Figures mbio.00693-26-s0001.docx Fig. S1 to S20.

Supplemental Tables mbio.00693-26-s0002.xlsb Tables S1 to S5.

ASM does not own the copyrights to Supplemental Material that may be linked to, or accessed through, an article. The authors have granted ASM a non-exclusive, world-wide license to publish the Supplemental Material files. Please contact the corresponding author directly for reuse.
