# Supplementary material for: Active prophages as key drivers of microbial adaptation in global soil ecosystems
Source: mBio. 2026 Jun 15;17(7):e00693-26. doi: 10.1128/mbio.00693-26 (PMC13343844; doi:10.1128/mbio.00693-26)
Supplement: Supplemental Figures — Fig. S1 to S20. [file mbio.00693-26-s0001.docx]

**Active prophages as key drivers of microbial adaptation in global soil ecosystems**

Chaofan Ai^1^, Xiang Tang^1*^, Haoxiang Han^1^, Yuqi He^1^, Hongbo Zhang^1^, Chen Liu^1^, Hanpeng Liao^1*^, Shungui Zhou^1^

^1^ Fujian Provincial Key Laboratory of Soil Environmental Health and Regulation, College of Resources and Environment, Fujian Agriculture and Forestry University, Fuzhou, 350002, China.

*Corresponding author:

Xiang Tang (tang61@fafu.edu.cn), ORCID: 0000-0001-6764-3447

Hanpeng Liao (liaohp@fafu.edu.cn), ORCID: 0000-0001-7539-2668


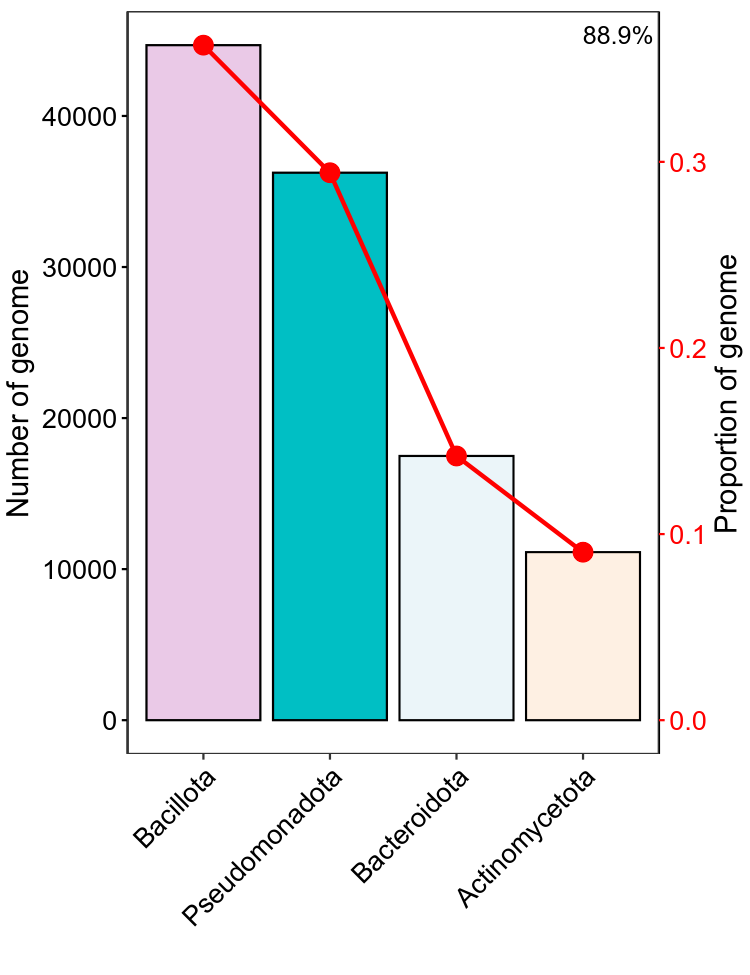


Fig. S1 Number and proportion of major bacterial genomes, with the four major phyla *Bacillota*, *Pseudomonadota*, *Bacteroidota* and *Actinomycetota* accounting for 88.9% of all bacterial genomes.


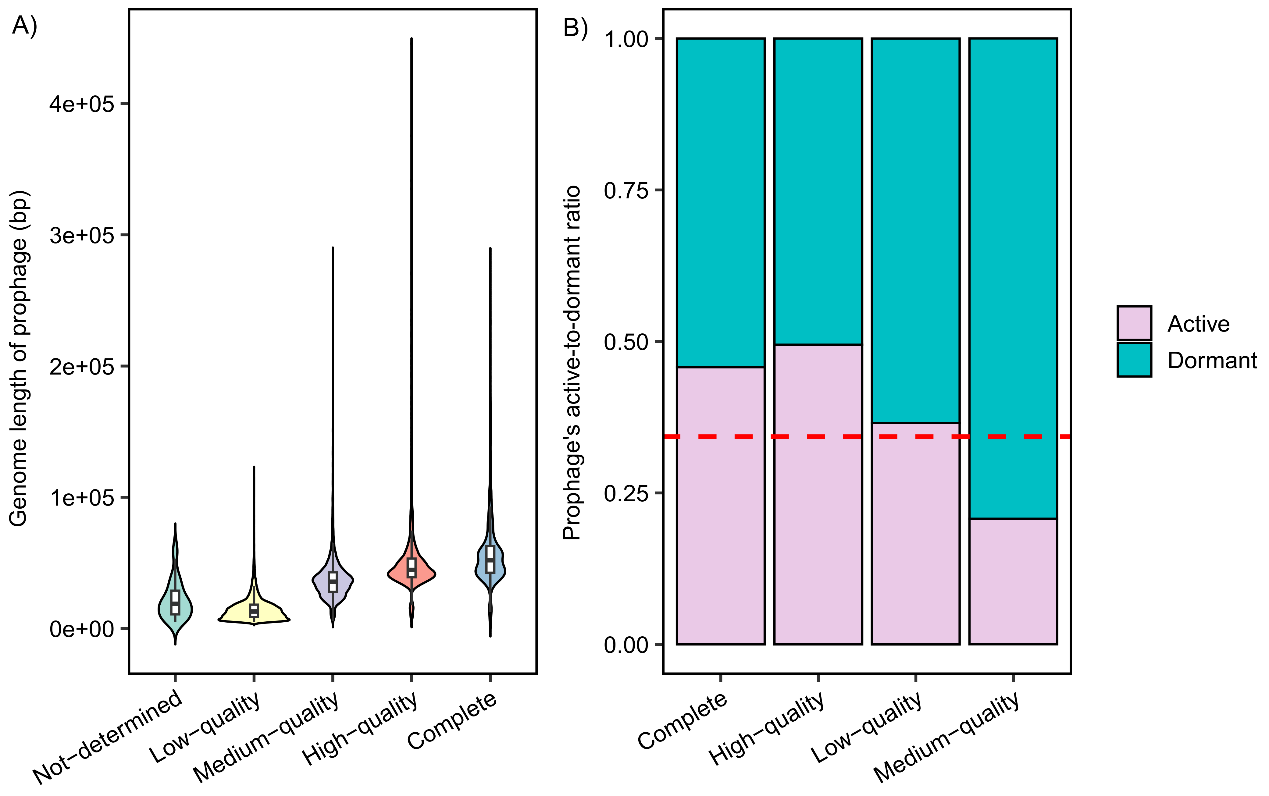


Fig. S2 Genome quality distribution of prophage. **A)** Genomic quality of prophage and corresponding genome lengths. **B)** Proportion of active and dormant in different genomic quality of prophage. The dotted line represents the mean proportion of active prophage across all genomic quality.


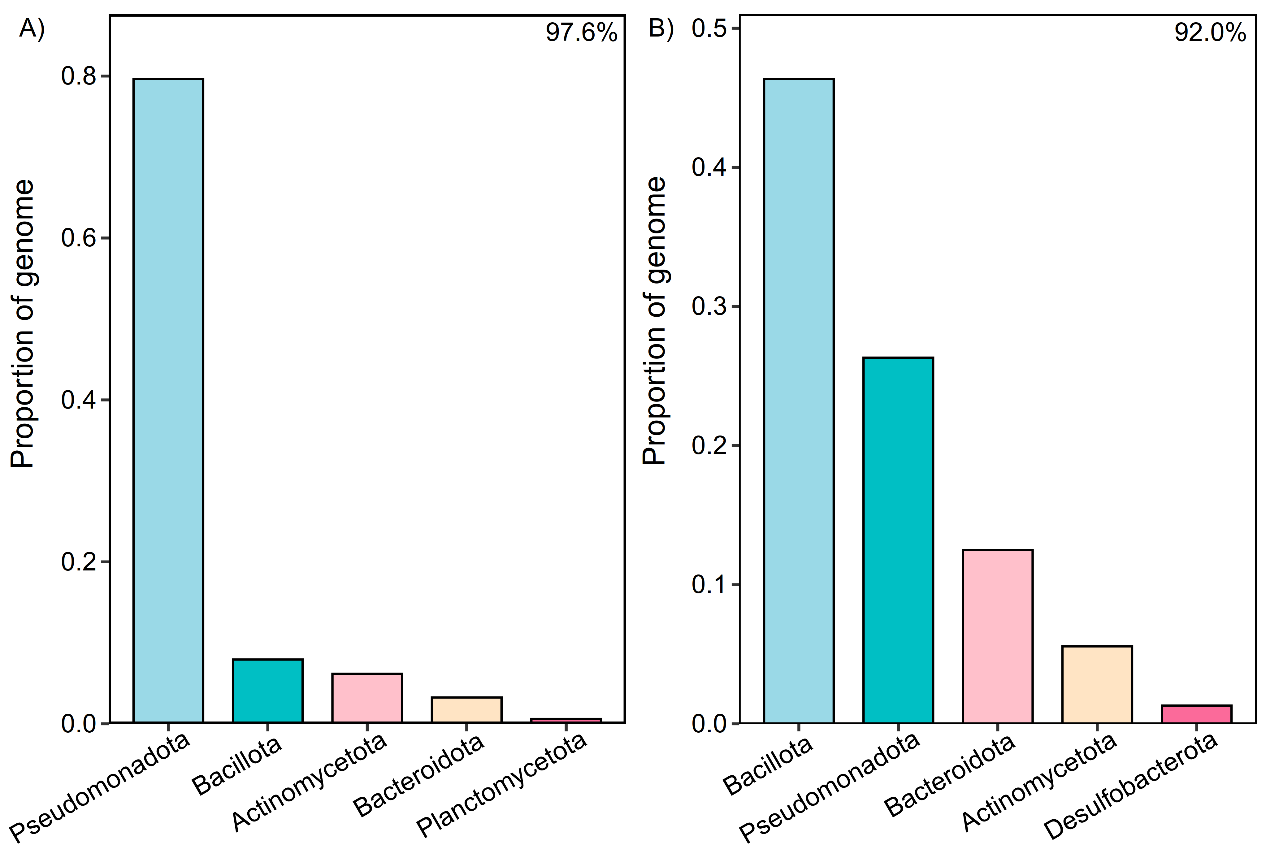


Fig. S3 The distribution of host bacteria for **A)** active and **B)** dormant prophages.


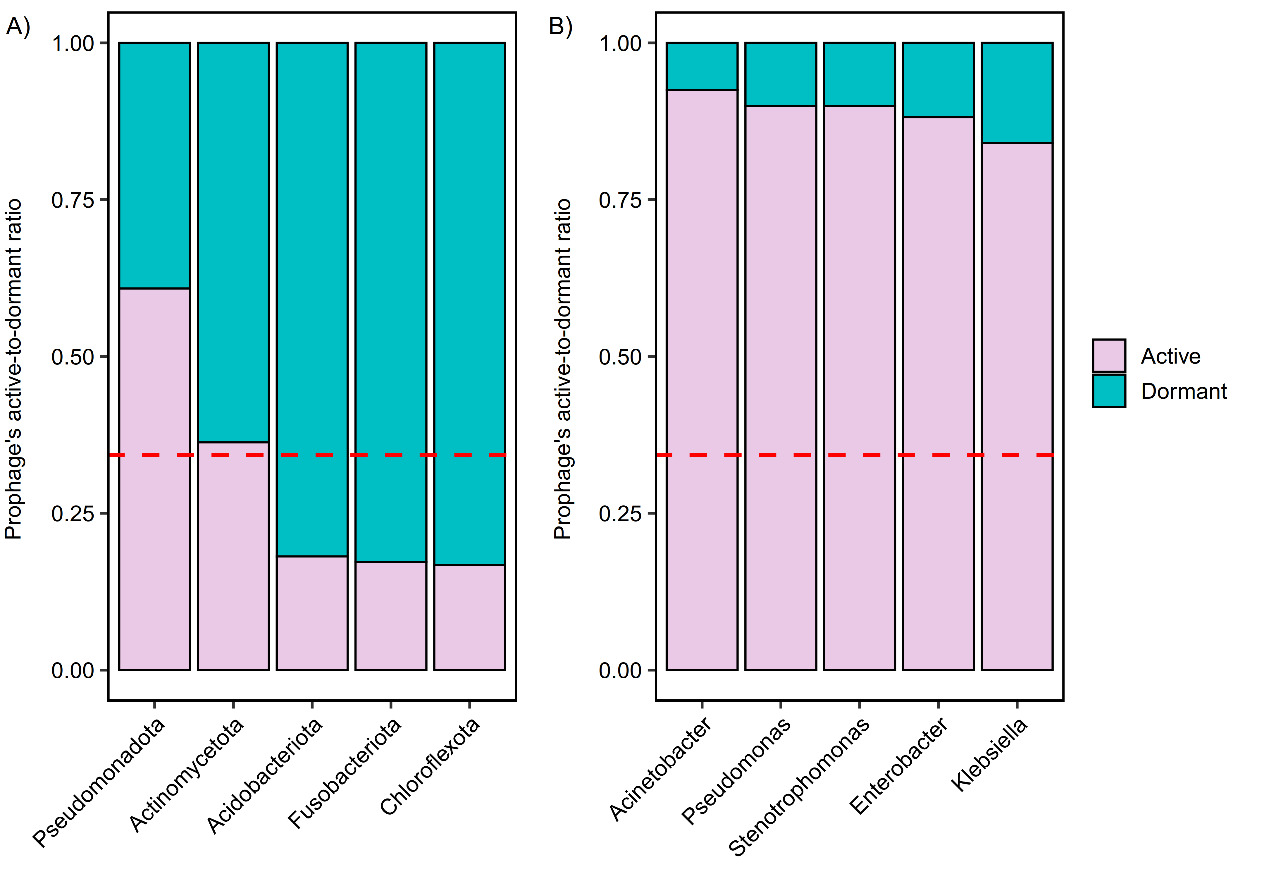


Fig. S4 The proportion of active and dormant prophages in different bacteria at phylum and genus level. **A)** The top 5 bacteria at phylum level were selected to calculate the proportion activity and dormant prophage. The dotted line represents the mean proportion of active prophage across all bacterial phylum **B)** The top 5 bacteria at genus level were selected to calculate the proportion activity and dormant prophage. The dotted line represents the mean proportion of active prophage across all bacterial genera.


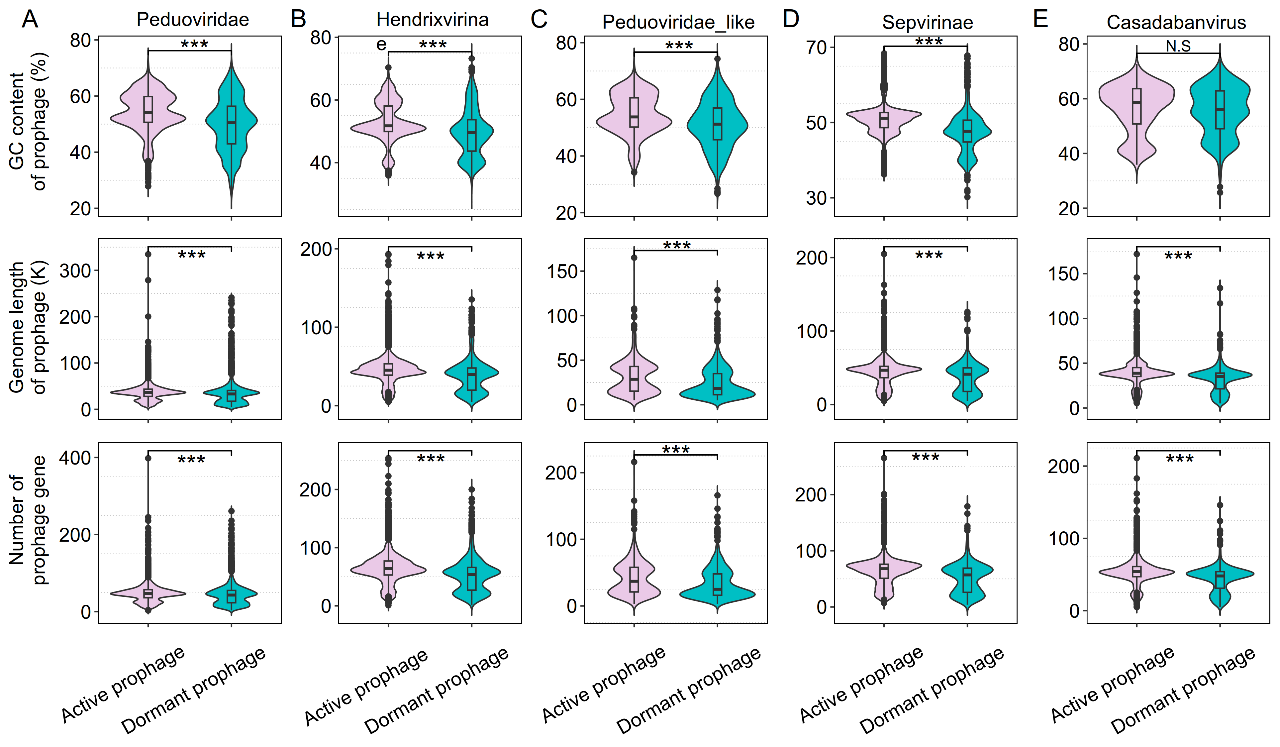


Fig. S5 Comparison of genomic features (GC content, genome length and number of genes) of active and dormant prophage. The top five prophages (*Peduoviridae*, *Hendrixvirina*, *Peduoviridae_like*, *Sepvirinae*, and *Casadabanvirus*) at family level were selected for genomic characterization comparison. Box plots show the 25th–75th percentiles, whiskers indicate the minimum and maximum values, and the midline represents the median. Student's t-test were applied when data followed a normal distribution, whereas non-parametric alternatives (Kruskal–Wallis and Wilcoxon tests) were used otherwise, and the significance levels are indicated by *** (*P* < 0.001) and N.S (*P* > 0.05).


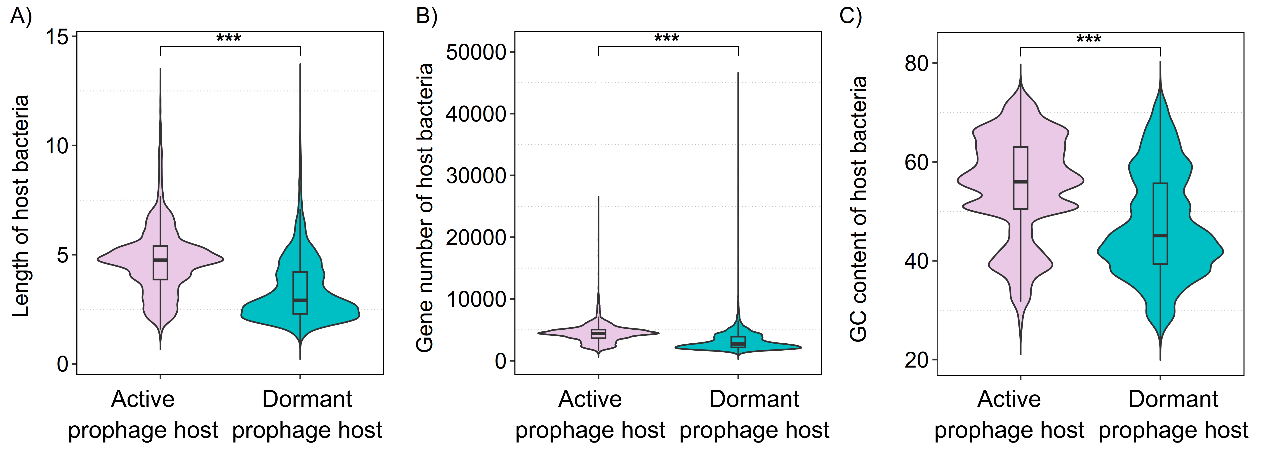


Fig. S6 Comparison of genomic characteristics of host bacteria between active and dormant prophages. Violin plot represents **A)** the length of genome, **B)** gene number, and **C)** GC content of active and dormant prophage. Box plots show the 25th–75th percentiles, whiskers indicate the minimum and maximum values, and the midline represents the median. Student's t-test were applied when data followed a normal distribution, whereas non-parametric alternatives (Kruskal–Wallis and Wilcoxon tests) were used otherwise, and the significance levels are indicated by *** (*P* < 0.001).


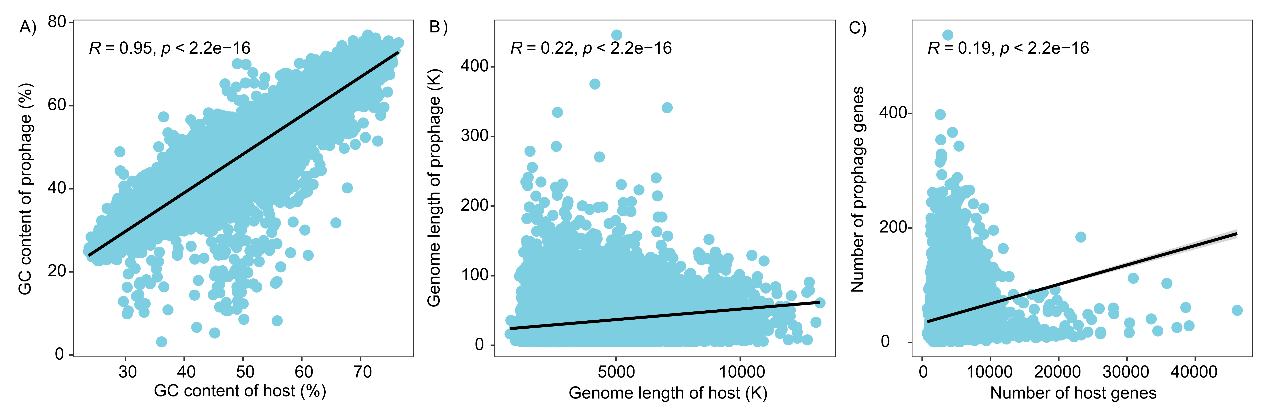


Fig. S7 The relationship between prophages and their hosts genomic features. **A)** The positive correlations between the GC content of prophage and host bacteria. **B)** The positive correlations between the genome length of prophage and host bacteria. **C)** The positive correlations between the gene number of prophage and host bacteria. The shaded area shows 95% confidence interval around the fitted mean line.


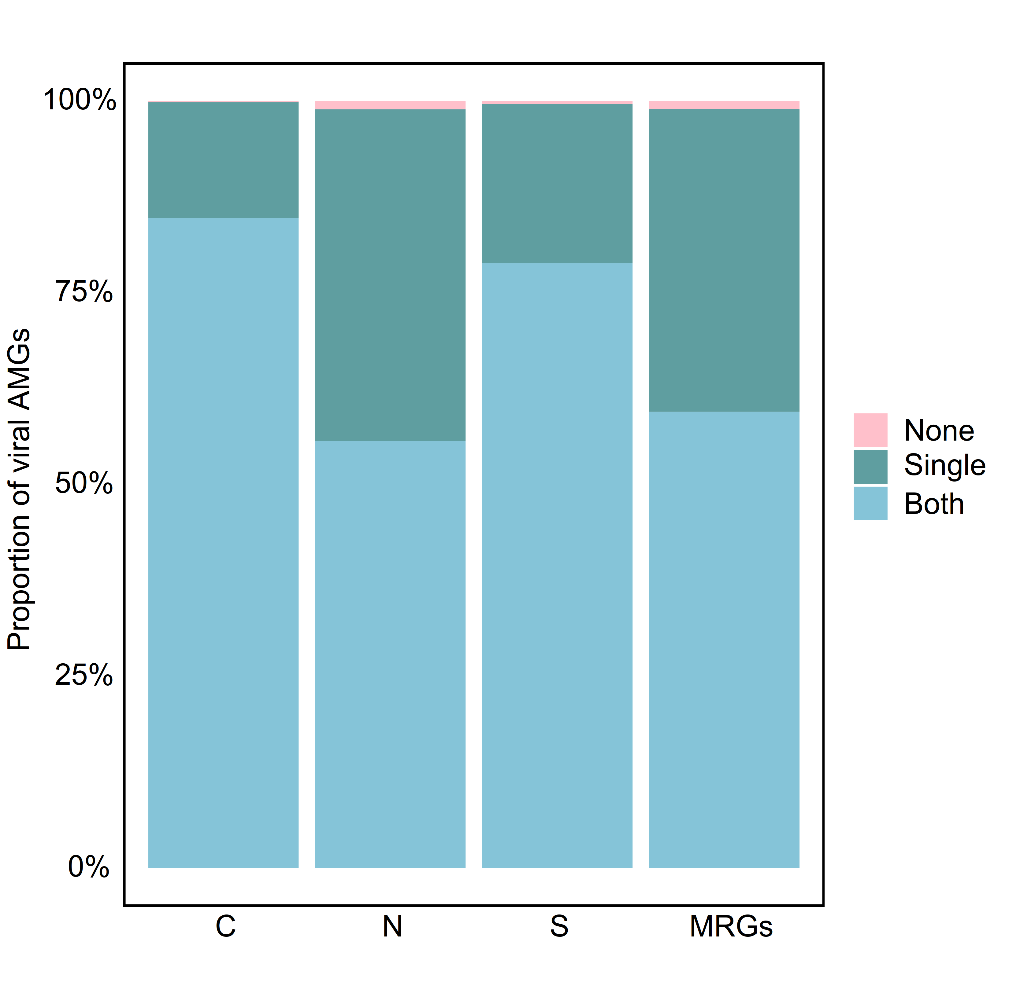


Fig. S8 Analysis of the distribution patterns of viral genes that flank CAZyme-related genes, nitrogen-related genes sulfur-related genes and metal resistance-related genes within phage genomes. The presence of viral-like genes within 20 genes upstream or downstream of each pCAZyme gene was identified using VirSorter2. “Both” indicates viral genes located on both sides, “Single” denotes viral genes on only one side, and “None” signifies the absence of viral genes on either side.


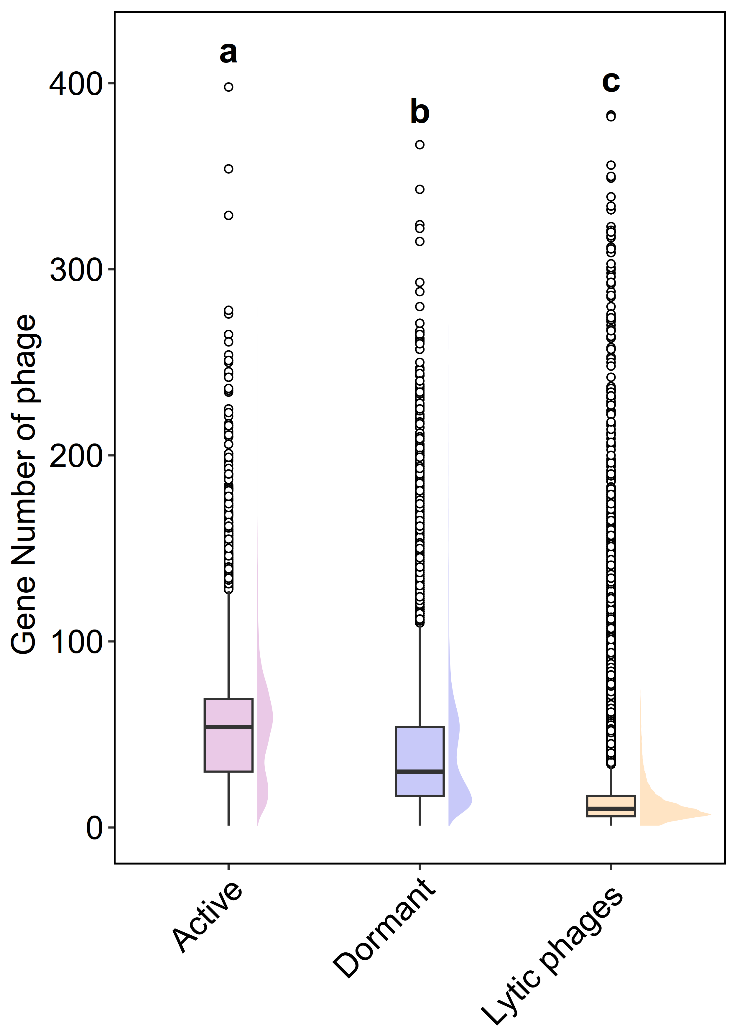


Fig. S9 Comparison of active, dormant and virulent bacteriophages carrying genes. All the significant differences between active and dormant prophage were determined by two-sided Wilcoxon rank-sum tests, and the significance levels are indicated by *** (*P* < 0.001).


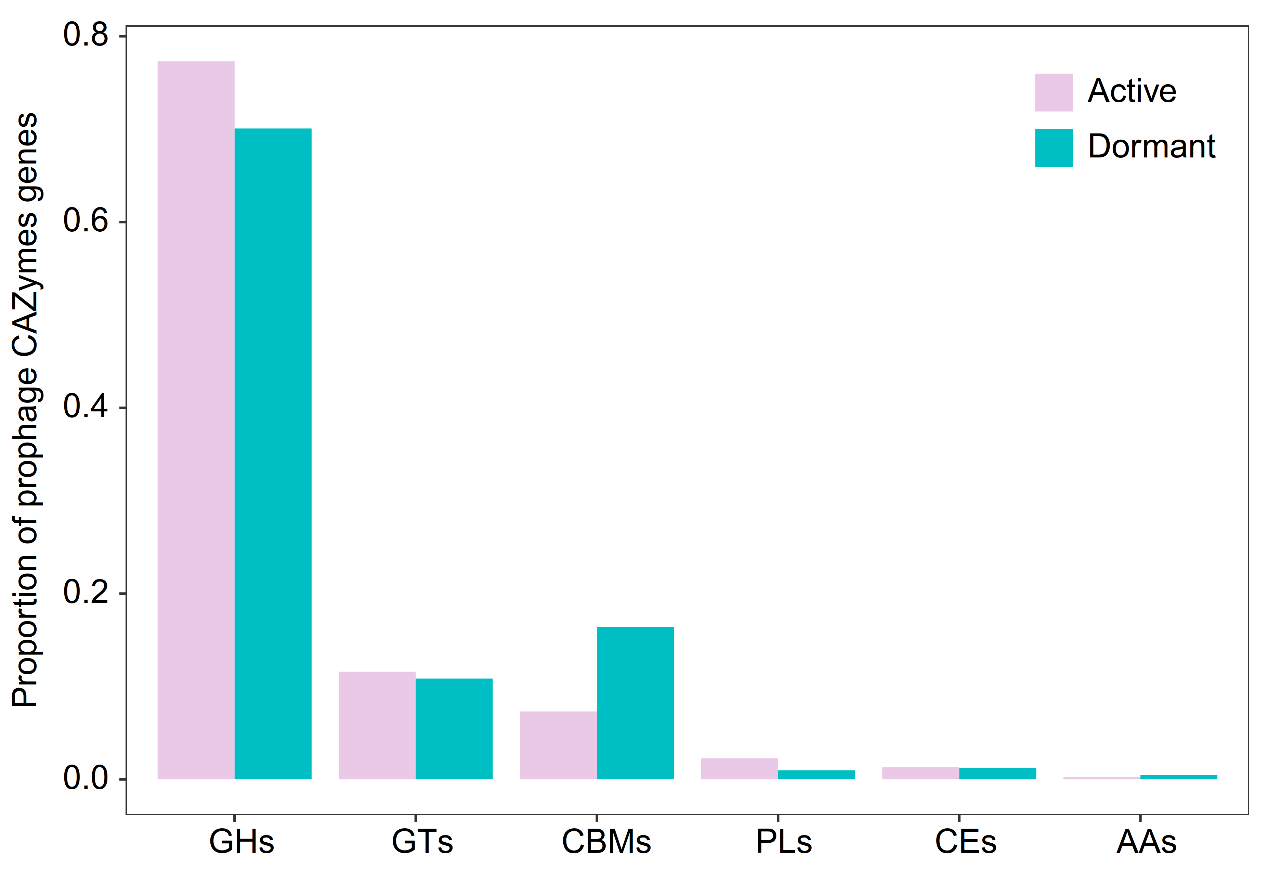


Fig. S10 The proportion of CAZyme genes encoded in active and dormant prophage genomes.


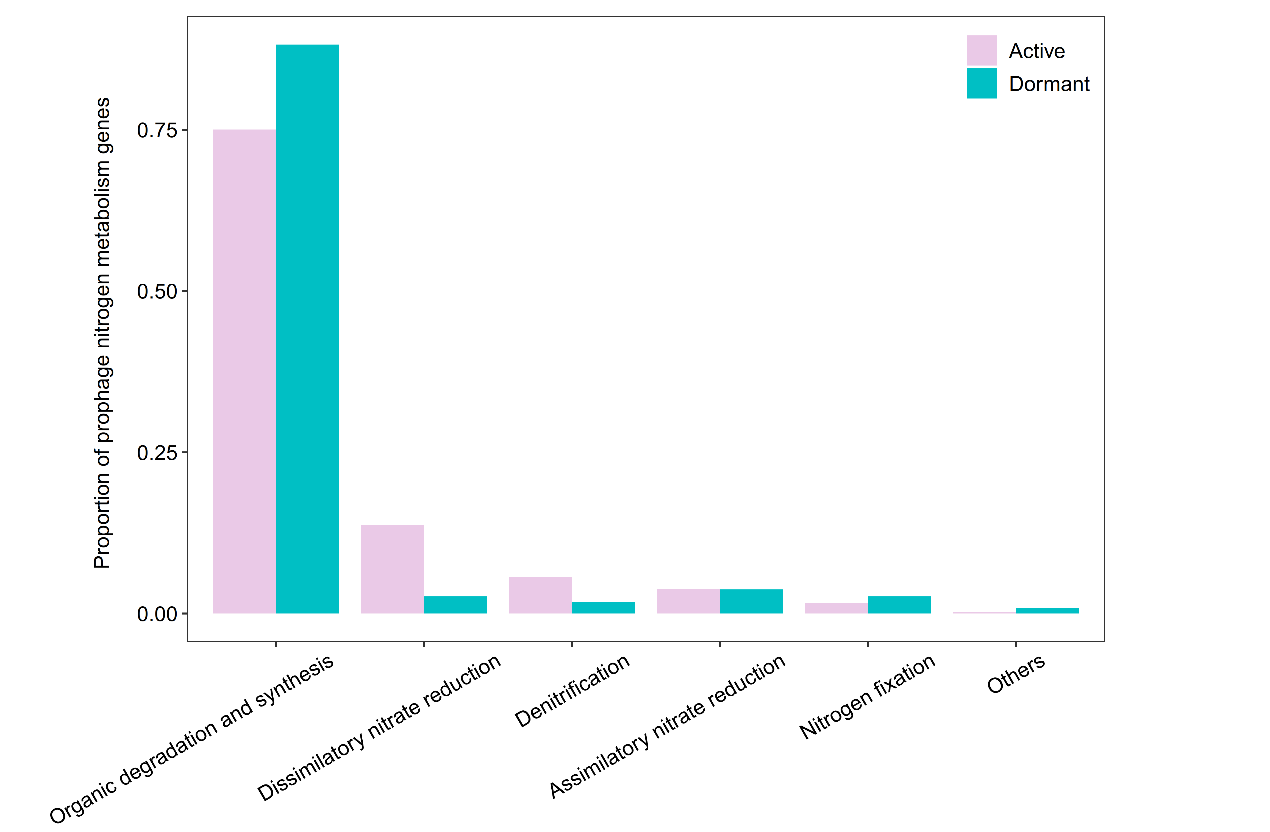


Fig. S11 The proportion of nitrogen metabolism genes encoded in active and dormant prophage genomes.


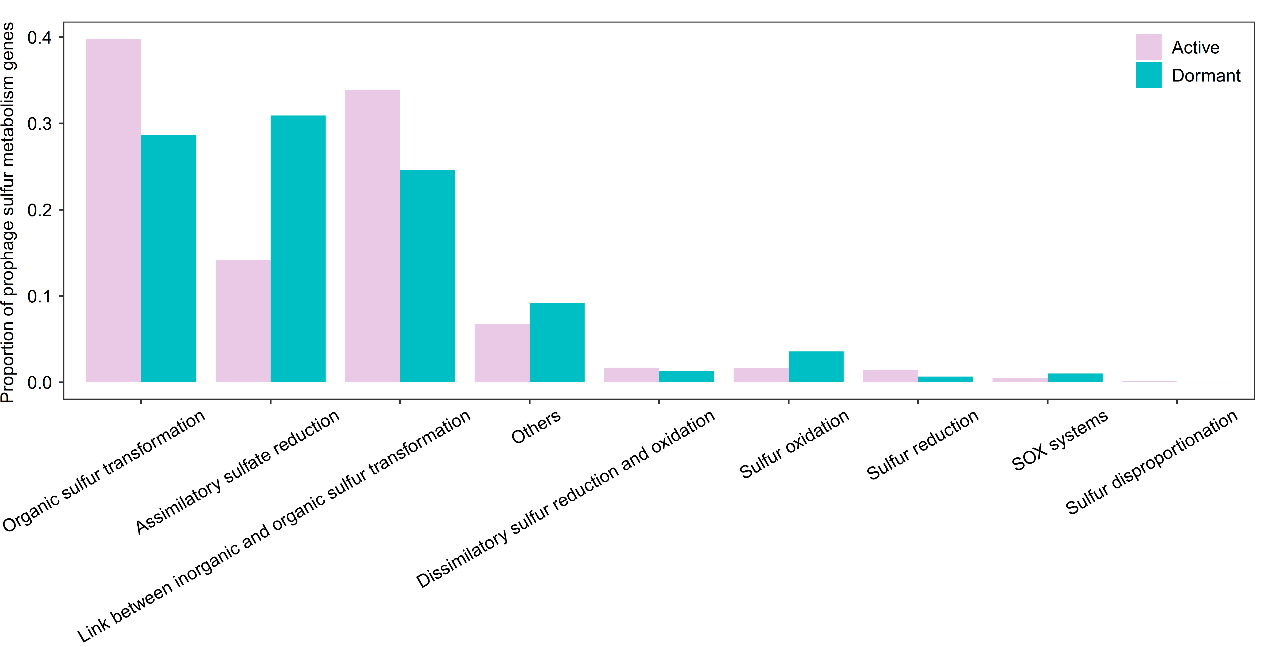


Fig. S12 The proportion of sulfur metabolism genes encoded in active and dormant prophage genomes.


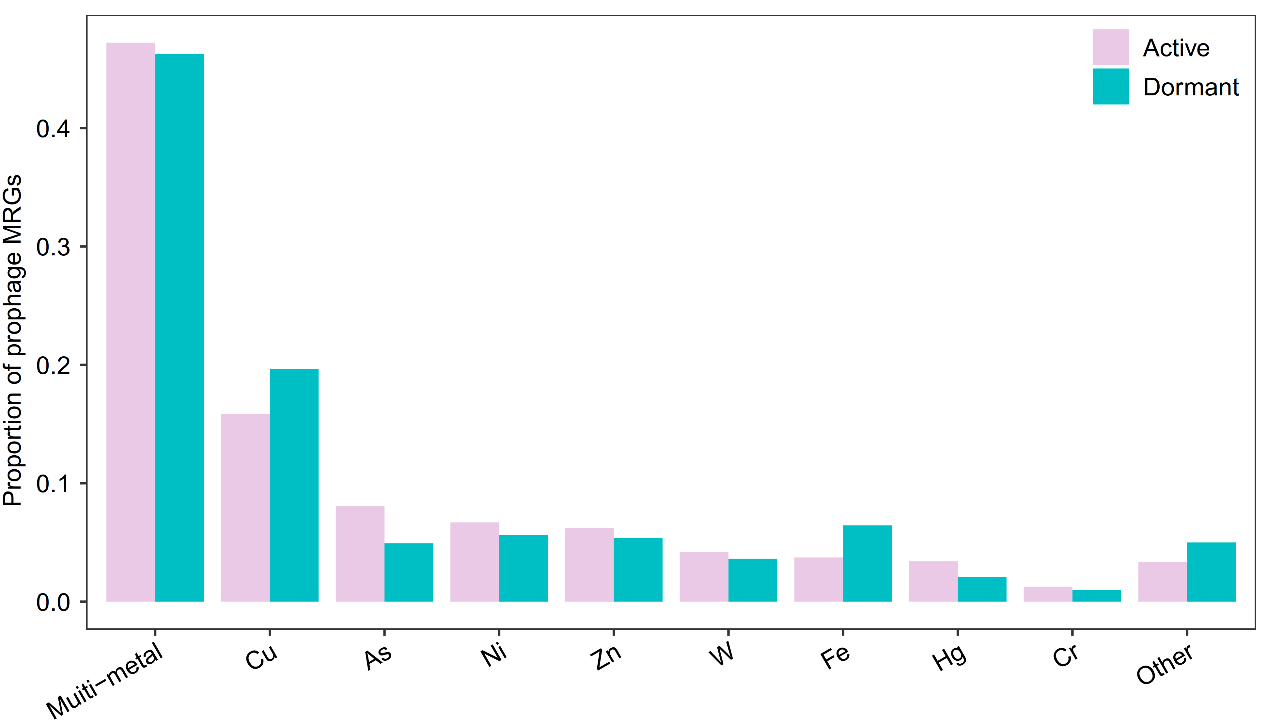


Fig. S13 The proportion of metal resistance genes encoded in active and dormant prophage genomes.


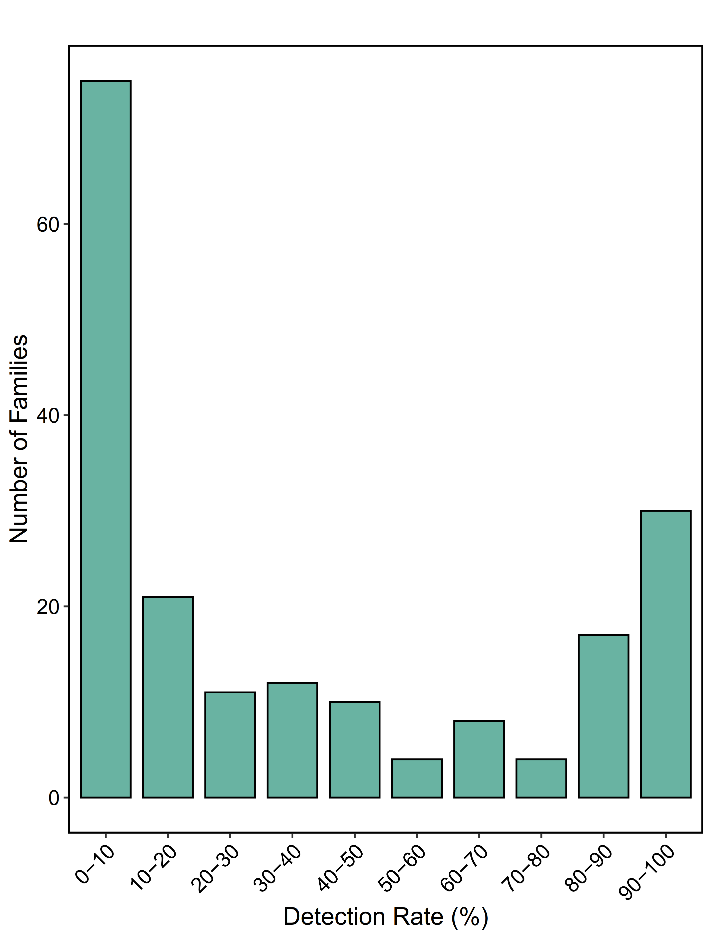


Fig. S14 The detection range of different viral families across global soil samples (n = 3,749). This data was calculated based on the abundance of prophages in the soil metagenome.


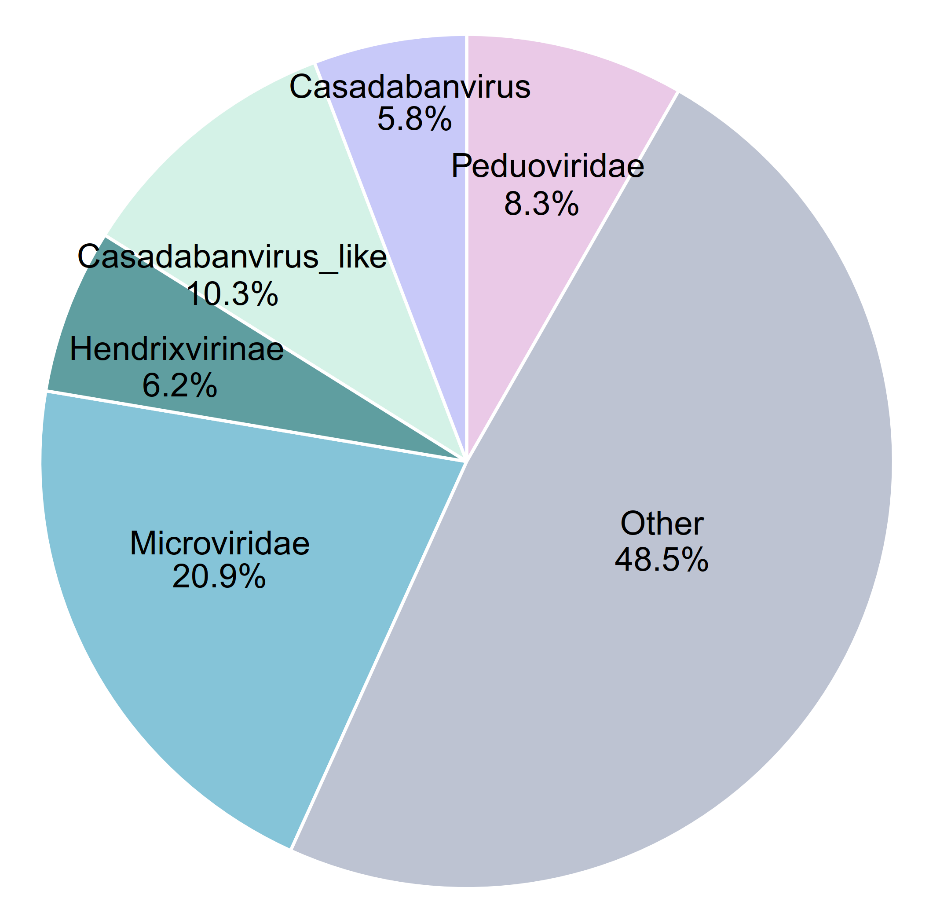


Fig. S15 The abundance proportion of dominant viral families in global soil. This data was calculated based on the abundance of prophages in the soil metagenome.


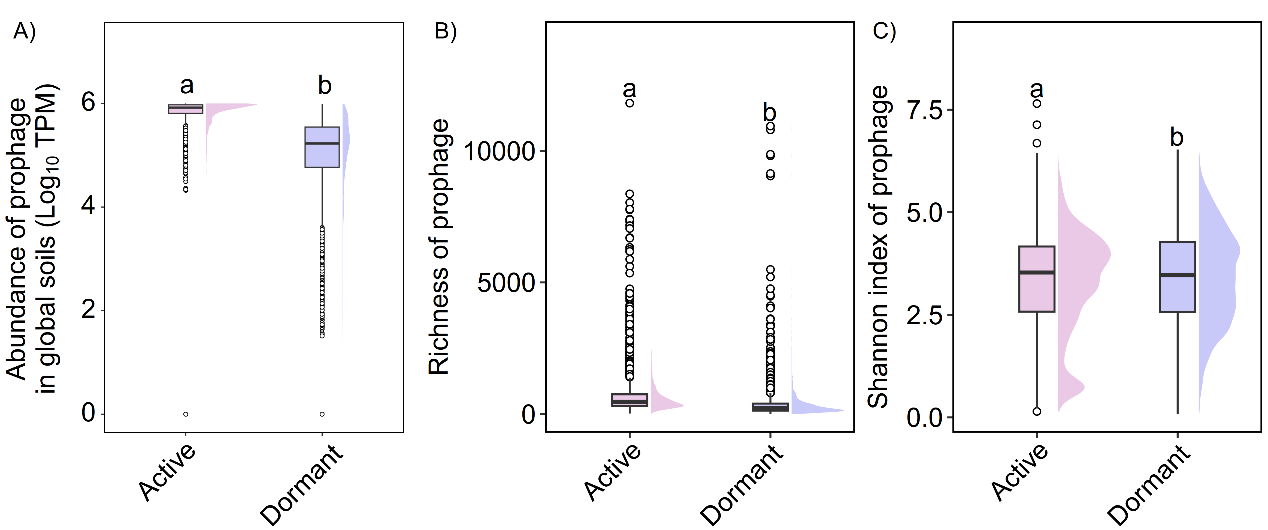


Fig. S16 Comparison of active and dormant prophages in global soils. **A)** abundance, **B)** Richness and **C)** Shannon index of active and dormant prophages across global soils. Box plots encompass 25-75th percentiles, whiskers show the minimum and maximum values, and the midline shows the median. Student's t-test were applied when data followed a normal distribution, whereas non-parametric alternatives (Kruskal–Wallis and Wilcoxon tests) were used otherwise, and different lowercase letters indicate statistically significant differences at *P* < 0.05.


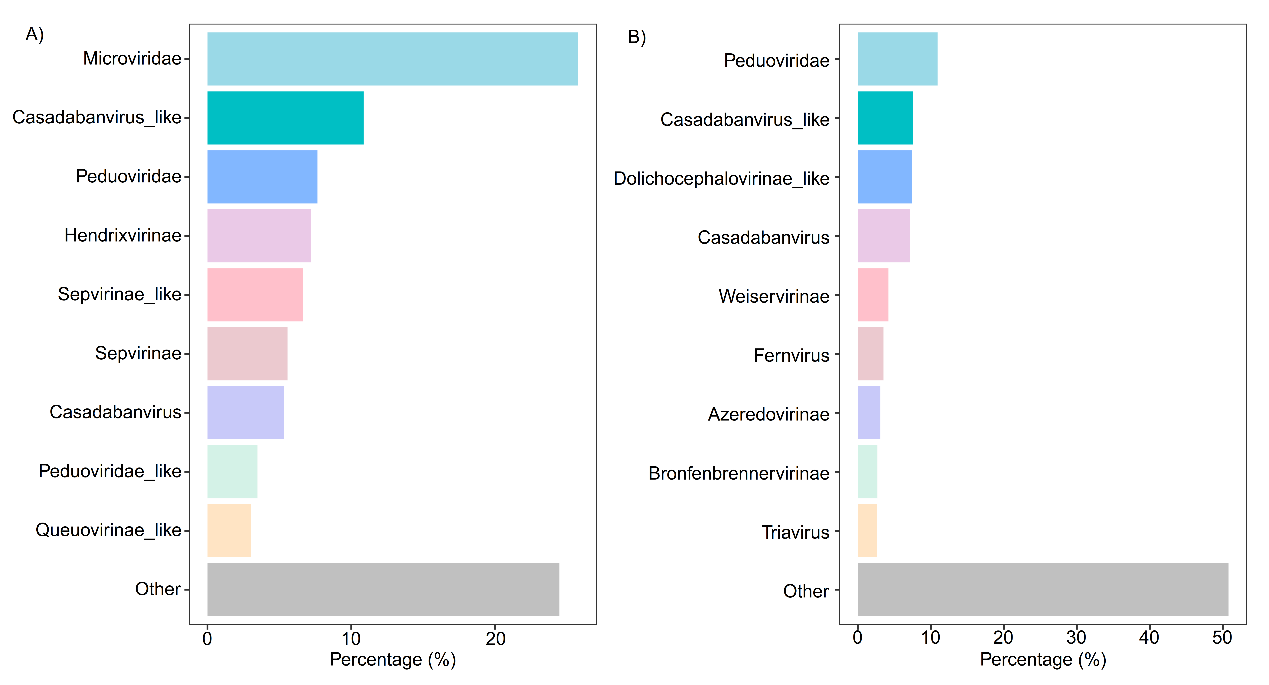


Fig. S17 Relative abundance of dominant **A)** active and **B)** dormant prophages in global soils. This data was calculated based on the abundance of prophages in the soil metagenome.


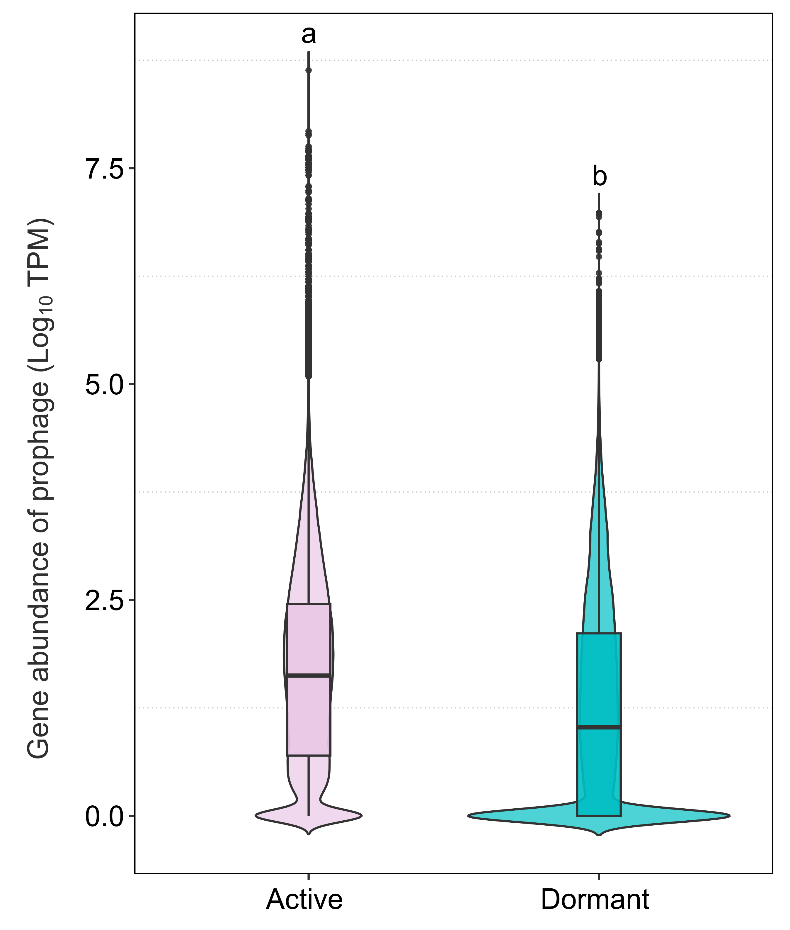


Fig. S18 Comparison of auxiliary metabolic genes encoded by active and dormant prophages in global soils. Box plots encompass 25-75th percentiles, whiskers show the minimum and maximum values, and the midline shows the median. Student's t-test were applied when data followed a normal distribution, whereas non-parametric alternatives (Kruskal–Wallis and Wilcoxon tests) were used otherwise, and different lowercase letters indicate statistically significant differences at *P* < 0.05.


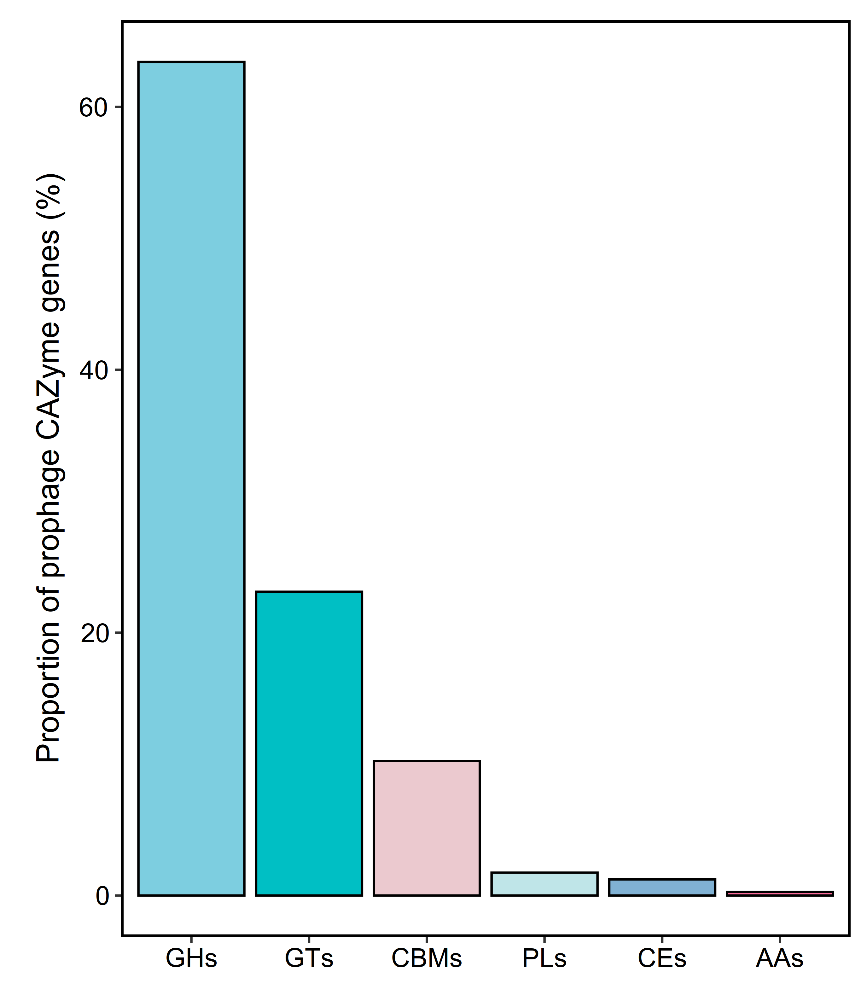


Fig. S19 Proportions of CAZyme genes encoded by soil prophages. This data was calculated based on the abundance of AMGs in the soil metagenome.


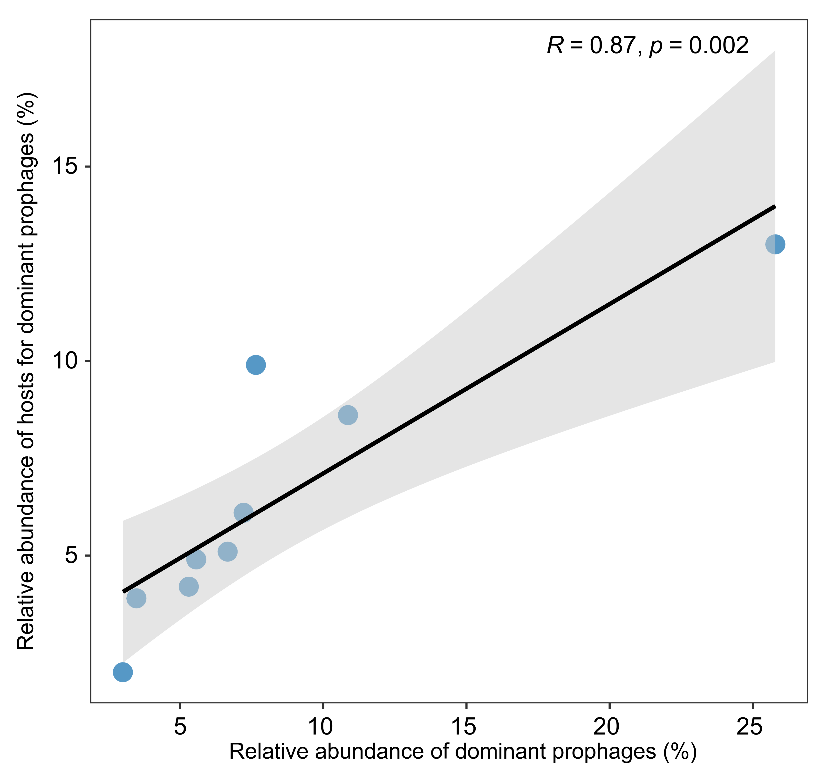


Fig. S20 The relationship between the relative abundance of dominant prophages and their hosts. The shaded area shows 95% confidence interval around the fitted mean line. This data was calculated based on the abundance of prophages and host in the soil metagenome.
